# Quantifying and explaining the rise of fiction

**DOI:** 10.1017/ehs.2025.10011

**Published:** 2025-07-14

**Authors:** Edgar Dubourg, Valentin Thouzeau, Quentin Borredon, Nicolas Baumard

**Affiliations:** École normale supérieure-PSL, Institut Jean Nicod, Paris, France

**Keywords:** fiction, cultural evolution, cultural ecology

## Abstract

We present a comprehensive analysis of the rise of fictions across human narratives, using large-scale datasets that collectively span over 65,000 works across various media (movies, literary works), cultures (over 30 countries, Western and non-Western), and time periods (2000 BCE to 2020 CE). We measured fictiveness – defined as the degree of departure from reality – across three narrative dimensions: protagonists, events, and settings. We used automatic annotations from large language models (LLMs) to systematically score fictiveness and ensured the robustness and validity of our measure, specifically by demonstrating predictable variations in fictiveness across different genres, in all media. Statistical analyses of the changes in fictiveness over time revealed a steady increase, culminating in the 20th and 21st centuries, across all narrative forms. Remarkably, this trend is also evident in our data spanning ancient times: fictiveness increased gradually in narratives dating back as far as 2000 BCE, with notable peaks of fictiveness during affluent periods such as the heights of the Roman Empire, the Tang Dynasty, and the European Renaissance. We explore potential psychological explanations for the rise in fictiveness, including changing audience preferences driven by ecological and social changes.

## Graphical abstract

**Figure:**
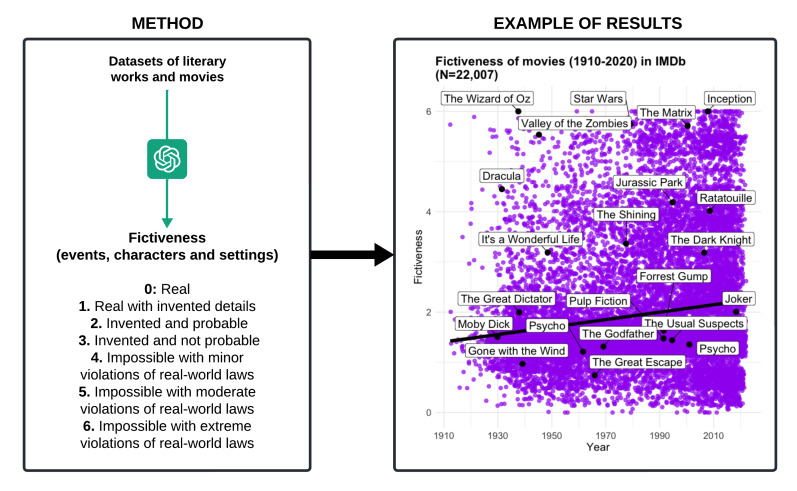

## Social media summary

Using 65,000 works, we track how fiction has grown more distant from reality across cultures and centuries.

## 1. Introduction

A fiction is a partially false story shared with the intent that the audience recognizes it as such (Currie, 1990; Genette et al., 1990; Schaeffer, 1999; Searle, 1975; Walton, 1993). This straightforwardly distinguishes fiction from other types of partially false stories, such as mistakes (where the sender is unaware that it is false), lies (where the sender intends to conceal that it is false), and religious myths (where the sender expects others to accept certain elements regardless of their truth value). Recognizing fiction therefore requires understanding not just the story’s content but also the author’s intent for it to be perceived as partly fabricated. At the psychological level, the recognition of fiction therefore involves metarepresentational skills (Sperber, 2000), most notably (1) pragmatic sense – a theory-of-mind mechanism that detects others’ intent and supports ostensive communication (Heintz & Scott-Phillips, 2023), but also (2) pretense – the ability to engage in ‘decoupled’ thought that separates imagined scenarios from reality (Leslie, 1987; Tooby & Cosmides, 2001) and (3) epistemic vigilance – the set of psychological mechanisms that assess the epistemic status of communicated information through prior knowledge or trust in the source (Mercier, 2017, 2020), which is crucial for recognizing fiction when the content is implausible or when a trusted source conveys an improbable story. All these mechanisms allow humans to recognize an imagined story as partially untrue without interpreting it as deceptive.

Because these basic mechanisms are part of the human evolved psychology, fiction has always been a possible form of storytelling within human groups. In other words, these psychological mechanisms enable humans everywhere and at any time to share stories, with the shared understanding that certain elements are not meant to be taken literally. In hunter-gatherer societies today, for instance, fiction is often signalled through pragmatic cues such as references to distant times (Sugiyama, 2017) or evidential expressions (Aikhenvald, 2004). In turn, people understand such stories as non-factual and don’t let the information within them guide their behaviours in real-life contexts (Bascom, 1965; Blurton Jones & Konner, 1976; Van Leeuwen, 2023). Wiessner’s study (Wiessner, 2014) on the !Kung Bushmen shows that fiction is also shared in specific contexts, notably around evening fires. Many stories from ancient societies were also recognized as being partly invented, providing clear evidence that fiction was an integral part of early storytelling practices. In *The Odyssey* for instance, Homer blends characters and settings believed to have existed by Greek audiences with *invented* situations and dialogues. Importantly, this was manifest at the time. Aristotle, who admired Homer’s ‘skillful lying,’ gave the example of Odysseus arriving in Ithaca while asleep: an event that is invented yet feels plausible within the story (Aristotle, 2011).

Despite the fact that, given human psychology, fiction was possible at all times, some researchers in literary theory and history have qualitatively observed what appears to be an increase in fiction throughout human history, in both Western (Bakhtin, 1984; Gallagher, 2006; Green, 2002; Paige, 2020) and non-Western (Postel, 2010) societies. This trend could manifest in two key ways. First, humans may increasingly share stories ostensibly marked as intentionally invented: a larger portion of the discourse humans consume would therefore consist of narratives that are fully understood to be intentionally invented rather than factual; this would reflect a rise in *fictionality*. Consider, for instance, the explicit labelling of many modern books as ‘fiction’. Alternatively, stories may progressively become more imagined and falser, that is, more distant from reality, through the introduction of more explicitly invented characters, events, or settings; this would represent an increase in *fictiveness*. Ancient works like *The Iliad* or *The Song of Rolland* often include known places and figures, making them much less fictive than many modern literary works like Tolkien’s expansive, fully imagined Middle-Earth.

Despite extensive qualitative work by literary theorists and historians suggesting an increase in fictionality over time, this claim remains speculative due to the lack of systematic empirical evidence (although see Piper, 2016). The challenge lies in the difficulty of quantifying fictionality consistently across different periods and cultures. Measuring fictionality in ancient texts is particularly challenging, as historical data that could reveal authorial intent or audience perception are often incomplete or unavailable. Moreover, the *intent*-based definition of fictionality, though conceptually clear, is difficult to operationalize due to the sophistication of the human pragmatic sense. For instance, some authors, while telling highly fictive stories set in non-existent worlds, added preface statements asserting their veracity. However, as argued by some scholars (Paige, 2020), such truth claims may not have been intended to be taken literally. This gap between *explicit* markers of (non-)fiction and *implicit* cues – often ironic or parodic – complicates the evaluation of historical audience attitudes. The issue is compounded in oral traditions, where culturally specific markers, such as tonal shifts (Scalise Sugiyama, 2024), are lost in transcription. Additionally, markers of fictionality are often context-dependent and shaped by local norms (Underwood & Sellers, 2012) – for example, the frequency of exclamation marks, which is highly associated with fictional discourse in the 19th-century English novel (Piper, 2016). These factors limit our ability to assess how fiction was signalled and perceived in the past.

In this article, we therefore turn to fictiveness as our focus. Unlike fictionality, fictiveness is more straightforward to quantify, as it involves assessing the content-level departure from reality, which is not dependent on subtle pragmatic cues. This approach has been successfully applied by Paige (2020), who differentiated between novels that featured well-known historical or mythical figures (i.e., ‘somebody’ novels, or Aristotelian novels, such as Œdipus) and those that introduced entirely new characters (described as ‘private individuals (…) unknown to readers before they open the book’; i.e., ‘nobody’ novels, such as Emma Bovary; Paige, 2020, p. 20). In his analysis of 17th- and 18th-century French literature, Paige found an increase in the prevalence of ‘nobody’ novels (p. 27), suggesting that the characters in these narratives were becoming increasingly *fictive*.

We choose to focus on fictiveness in our analysis not only because it is operationalizable but also because fictiveness and fictionality are, in any case, closely tied: the more a story introduces invented characters, events, or settings that significantly depart from reality (i.e., the more *fictive* it is), the clearer it becomes to audiences that the creator did not intend to deceive them (i.e., the more *fictional* it is) – the intuition being that more extreme departure from reality renders the story implausible as a lie, reinforcing its fictional status (Gallagher, 2006). Of course, the converse is not necessarily true: fictional stories can exhibit very low levels of fictiveness, as is often the case with recent autofiction, historical novels, or works of literary realism and naturalism. However, if highly fictive stories are consistently recognized as fictional, then tracking trends in fictiveness provides an informative proxy for understanding broader trends in fictionality over time: an increase in fictiveness likely reflects a rise in fictionality – because highly fictive works are most often recognized as fictional, even though not all fictional works are highly fictive.

Our contribution in this work is to propose a method to quantify how fictive a given story is and a systematic analysis of its presence and variation over time. Specifically, we analyse fictiveness conceived as a departure from reality in several large datasets of fictions, including movies, novels, and manga, both Western and non-Western, ancient and modern. Fictiveness is measured on three different narrative dimensions – events, protagonists, and settings – using scales that assess the degree of departure from reality, calibrated to the standards of the time and place to ensure contextual accuracy. Given the challenge of manual annotation, we employ large language models (LLMs), more specifically generative pretrained transformers (GPT), to annotate stories systematically, focusing on the fictiveness of each dimension while accommodating a wide array of historical and contemporary texts. This approach allowed for a scalable and standardized analysis of how the fictiveness of narratives change across time in distinct cultures.

## 2. Method

### 2.1. Datasets

**Figure 1.**
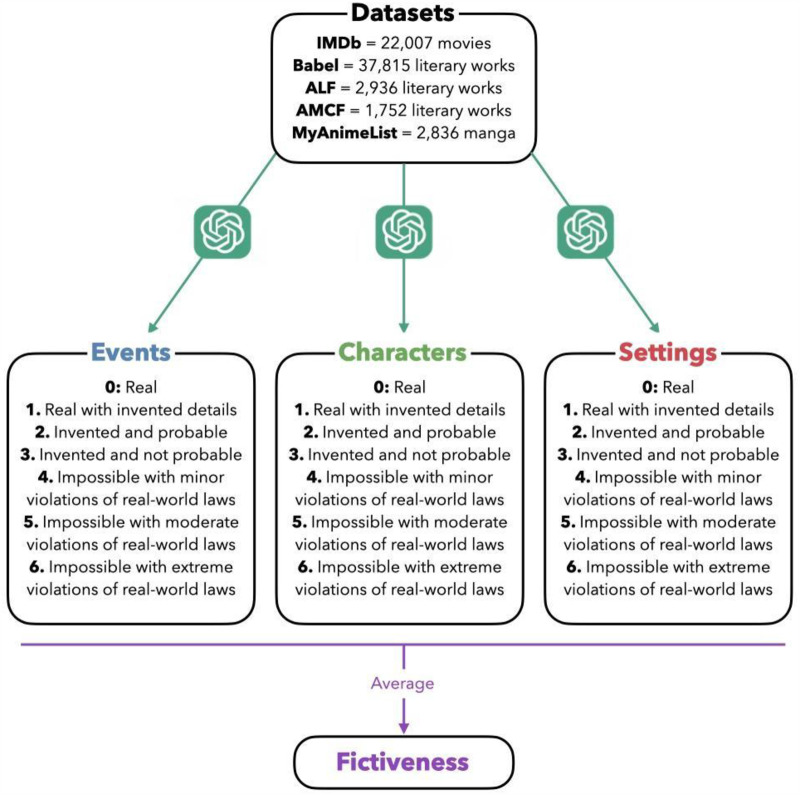
Data extraction and annotation process, with the minimal version of the scale (see SM for the full scales and the prompt).

To assess fictiveness across various narrative forms, we use five distinct datasets: IMDb, Babel, the Ancient Literary Fictions (ALF) dataset, the Ancient and Modern Chinese Fictions (AMCF) dataset, and MyAnimeList (see Fig. 1). Our sample of the IMDb dataset provides metadata on 22,007 movies released from 1914 to 2020. The Babel dataset is a new compilation of literary works sourced from Wikidata, an open-source knowledge base, which consists of 37,815 literary works spanning from 800 BCE to 2020 CE, encompassing over 30 languages. The ALF database, compiled by Baumard (2020), includes 2,936 literary fictional narratives spanning from 2000 BCE to 1800 CE, in 19 distinct geographical eras. The AMCF dataset, generated by Zhong et al. (2025), provides a collection of 1,752 Chinese fictional works from 400 to 2020, drawing on Wikipedia, Baidu Baike (a Chinese collaborative online encyclopedia), and manual extraction. Finally, MyAnimeList contains metadata on the 2,836 highest-rated manga series published between 1931 and 2020 (see Supplementary Material [SM] for more details about the datasets).

### 2.2. Scales

Fictiveness is a continuum, with some works making fewer departures from reality compared to other ones. Ancient Greek tragedies, like those featuring Œdipus or Antigone, drew from figures and events believed to have existed, as do many modern dramas, such as *The Crown* or *The Imitation Game*. In contrast, other stories feature fully invented characters. Early novels like Defoe’s *Robinson Crusoe* and Richardson’s *Pamela* presented characters and events that, while plausible, were completely invented, moving fiction away from reliance on history and myth. More contemporary fictional works often invent characters too, from plausible figures like Sherlock Holmes to fantastical, impossible ones like Superman. Some narratives even create entire fictional worlds, as seen in *One Piece* or *Lord of the Rings* for instance (Dubourg & Baumard, 2022b).

We distinguish fictiveness across three narrative dimensions: events (the actions within the story), protagonists (the characters driving the narrative), and settings (the locations and worlds in which the story unfolds). This partition is a standard approach in literary analysis. We hypothesized that each element can independently vary in its degree of departure from reality. Qualitative evidence suggests that stories can indeed make one aspect fictive while keeping others grounded in reality. For instance, literary novels very often introduce invented characters within completely realistic settings.

To quantify fictiveness, we therefore developed three scales to systematically assess how much a story’s events, protagonists, and settings, respectively, diverge from reality. These scales distinguish between elements that are real or believed to be real by the standards of the time and place (0-1), and those that are invented (2-6) with further distinctions based on the level of (un)plausibility and (im)possibility of the elements depicted (see Fig. 1). We specified in the prompt that the existence, plausibility, and possibility of the events, protagonists, and settings should be evaluated ‘by the standards of the time and place’, ensuring that the assessment takes into account the historical context in which the story is set, avoiding anachronistic judgments and allowing for a more accurate representation of how the narrative elements from ancient stories would have been perceived by contemporary audiences (see SM for the full scales and prompts).

### 2.3. Annotation

The main challenge in quantifying fictiveness has been the manual nature of annotation (Paige, 2020), which requires extensive expertise, particularly when analysing stories that few people are familiar with. Conducting a large-scale manual annotation would require a high number of experts to evaluate texts based on our scales, as knowledge of obscure or lesser-known works is often dispersed across people’s minds and their textual productions – specialized books, theses, academic publications and other sources. Yet this knowledge has been increasingly transferred to the internet through the digitization of these texts.

One issue that remained was the challenge of extracting consistent data from this scattered and heterogeneous body of online information. Here, LLMs, which are trained on a wide array of texts from the internet and can synthesize information efficiently, provided a solution. They can help gather and standardize annotations, even when the sources vary widely in format and style. Indeed, LLMs have already shown significant promise in annotating cultural data at scale (Abdurahman et al., 2023; Brown et al., 2020; Ding et al., 2023; Grossmann et al., 2023). Studies have demonstrated that GPT’s accuracy in data annotation tasks often exceeds both that of other annotation methods (Bongini et al., 2023; Pei et al., 2023; Rathje et al., 2023) and that of human coders (Gilardi et al., 2023). Its zero-shot learning ability enables it to perform well without retraining (Bongini et al., 2023; Ding et al., 2023; Kuzman et al., 2023; Pei et al., 2023), even in specialized domains (Fink et al., 2023; Savelka et al., 2023). Crucially, Bongini et al. (2023) emphasize that GPT does not need retraining to incorporate new knowledge about cultural artifacts to annotate them, thanks to its memorization capacity from observing millions of tokens during its original training. This characteristic is particularly advantageous for our work, allowing scalable annotation without additional model fine-tuning.

Previous studies have successfully used LLMs to annotate cultural products, including video games (Dubourg & Chambon, 2025) and literary works (Dubourg et al. 2025). In the video game project, GPT-generated scores on agency and exploration correlated with players’ related dimensions as pre-registered, strongly supporting the validity of the annotations. In the study of imaginary worlds, GPT was used to identify such *imaginary* worlds across a large corpus of ancient and modern literary works. Its identification was consistent with prior manual annotations, as well as with two other computational approaches: one based on embeddings and another using a random forest classification algorithm trained on manually annotated data (Dubourg, Thouzeau, Baumard, et al., 2023). Once identified, specific features of these imaginary worlds were also estimated by GPT (e.g., their size, their consistency). We were then able to compare these estimates with features already annotated by a literary historian in a comprehensive work (Wolf, 2013). GPT’s estimates of world size showed strong convergent validity, as they closely matched Wolf’s typology distinguishing between world types, such as islands, countries, planets, and universes. At the same time, GPT’s annotations also demonstrated discriminant validity. For example, GPT estimated whether the characters in these stories actively explored their worlds – an independent feature that should not strongly correlate with Wolf’s typology of world size. The fact that GPT’s annotations for character exploration did not simply mirror Wolf’s size classifications supports the interpretation that GPT was able to capture different dimensions of fictional worlds, not redundantly encoding the same information. In the present project on fictiveness, beyond checking that our scores correlate with genres as expected (see 3.1.), there is no comparable metadata or large-scale manual annotation feasible: reading or watching hundreds of works would be prohibitive. Here, we rely on the tool’s demonstrated capacity to provide annotations at scale.

Our automatic annotation method (see Dubourg, Thouzeau, Baumard, et al., 2023 for a step-by-step outline) therefore uses GPT to annotate the stories in our datasets, leveraging its extensive knowledge base. For each work, we provide only minimal identifying information – typically the title, date, and author when available – relying on the model’s training data to retrieve relevant background knowledge. Our prompt starts by specifying the referent, directing the LLM to focus on a particular aspect of the narrative – main protagonists, events, or settings; then it asks the LLM to evaluate the fictiveness of a given title with the specified scale and to provide a brief explanation of the evaluation (see SM and Fig. 1). We also asked the model to assign ‘NA’ (for ‘not applicable’) to unfamiliar texts that may not be well described within the LLM’s dataset. With three queries to GPT for each work (one for each referent: protagonists, events, and settings) and over 65,000 different works in our analysis, we therefore conducted a total of more than 195,000 distinct queries to GPT. To compute an overall fictiveness score, we averaged the scores of all three referents for each work.

Here is the standard prompt: ‘Evaluate the literary work based on the specified referent using the following scale. [Insert Scale]. You must assess the degree of invention, probability, and possibility according to the worldview and beliefs of people in the historical period of the work, without applying modern standards for ancient works. You must evaluate only [the main protagonists/the events/the settings] and ignore any other elements of the work. Provide a brief explanation and conclude the explanation with the score formatted as Score = followed by the numerical value, with no text or symbol after the score. If you are unfamiliar with the work, assign the score as NA. The work is: [Insert title] by [Insert author] written in [Insert date].’

### 2.4. Output

In our analysis, each work in the five datasets – IMDb, Babel, ALF, Baidu, and Manga – was annotated with three outputs from GPT for the fictiveness of each referent – protagonists, events, and settings. Each work was also assigned three fictiveness scores, each ranging from 0 to 6 (see Fig. 1). To have a first estimation of the reliability of these automatic annotations, we manually reviewed dozens of randomly selected works from each dataset. The model’s annotations were consistent with our expectations.

For instance, one of the randomly selected movies was *Shriek of the Mutilated* (1974). The characters are portrayed as ‘ordinary humans without any supernatural abilities’, which makes their existence entirely plausible by the standards of the 1970s, leading to a score of 2. However, the film’s events are centred around a Yeti – a mythical creature – which involves ‘a significant departure from any credible historical or scientific evidence’, and thus these events are given a score of 5 for involving moderate violations of real-world laws. The setting, while based on a real geographical location, contains ‘invented details’, resulting in a score of 1. The average fictiveness score for this work is therefore 2.67. This example illustrates that the fictiveness of each referent – characters, events, and settings – can vary significantly within a single work.

**Figure 2.**
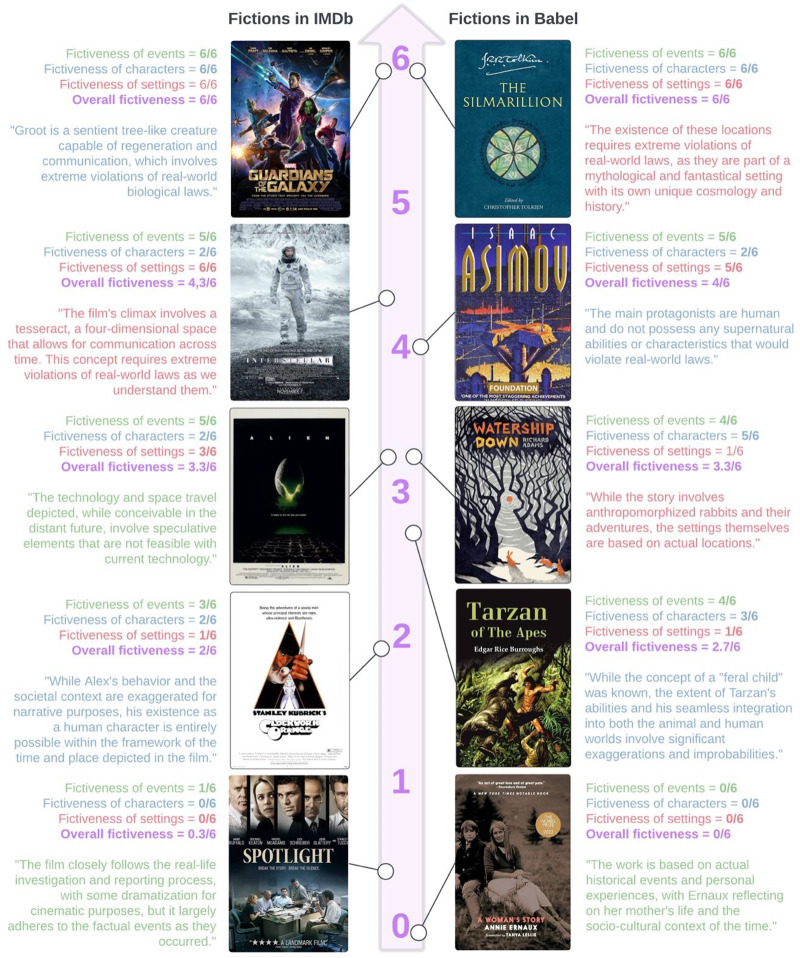
Examples of films (from IMDb) and literary works (from Babel), alongside their fictiveness scores for each referent, the overall (averaged) fictiveness score, and a selected excerpt from GPT’s generated output for one chosen referent (indicated by the colour of the text).

All the GPT-generated scores and justifications for each annotated work across our five datasets are available for review on our Open Science Framework (https://osf.io/kdtzf/; see Fig. 2 for supplementary examples).

Further validity and robustness checks are detailed in the SM. These include an analysis of annotation convergence between GPT-4 and another LLM (DeepSeek). While we observed moderate to strong agreement across most datasets, convergence was notably weaker for the MyAnimeList dataset. Additionally, we examined the distribution of works flagged as ‘NA’ (i.e., unfamiliar) by GPT and confirmed that these cases represented a small proportion of the data and were broadly distributed over time rather than clustered in specific periods. It was not the case for MyAnimeList. Given the lower convergence with DeepSeek and inconsistent annotation performance over time, we ultimately chose to exclude the MyAnimeList dataset from the final temporal analyses. However, it is worth noting that in our manual reviews, GPT’s annotations were more accurate than DeepSeek’s.

## 3. Analysis and results

The annotated datasets and the annotation and analysis script are all available on OSF (https://osf.io/kdtzf/).

### 3.1. Fictiveness varies across referents, media, and genres

**Figure 3.**
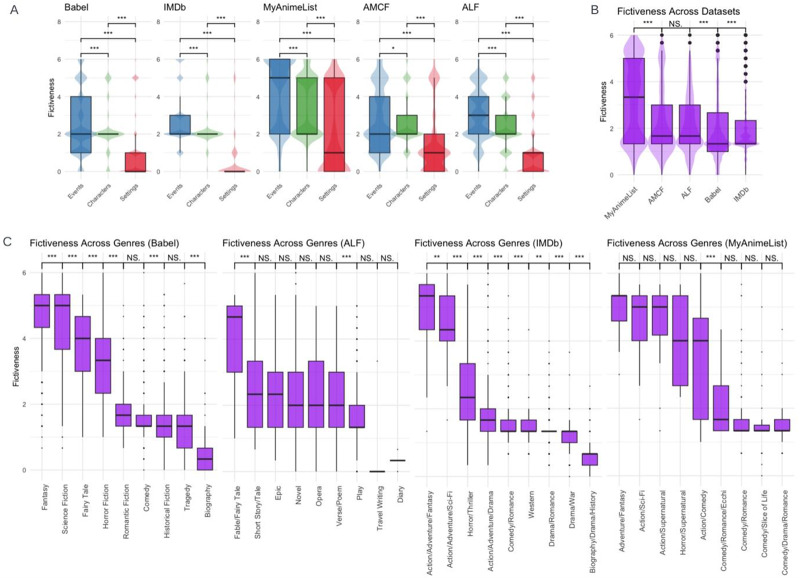
(A) Comparisons of fictiveness scores across referents in all five datasets. (B) Comparisons of fictiveness across datasets. (C) Comparisons of fictiveness across genres in four datasets where genres were available. In each graph, genres are ordered from higher to lower average fictiveness. For displaying significance, the overall fictiveness of adjacent genres is compared using a *t*-test (see SM for full statistics).

Across all the datasets, the scores for fictiveness consistently show a pattern where events tend to be more fictive than characters, and characters are, in turn, more fictive than settings (Fig. 3A to Fig. 3E). This pattern aligns with the idea that inventing entire worlds requires the invention of characters and settings, contributing to a narrative that is overall more distant from reality.

Using *t*-tests, we found variations of overall fictiveness between the datasets (Fig. 3B), with manga (MyAnimeList) showing higher levels of fictiveness compared to literary works (AMCF, ALF, and Babel). This aligns with qualitative observations: manga like *One Piece* or *Dragon Ball* are known for their fantastical worlds, extraordinary characters, and surreal events. This trend could be partly explained by the target audience of manga, which largely consists of young people who show greater interest in imaginary worlds and scenarios due to their heightened levels of curiosity (Dubourg, Thouzeau, de Dampierre, et al., 2023). Movies showed the lowest levels of fictiveness. This might be explained by budget constraints. While writing or illustrating highly fictive worlds and events does not require extra costs, bringing them to life in cinema requires substantial resources for special effects and set design (Tinits & Sobchuk, 2020).

For four datasets – IMDb, Babel, ALF, and MyAnimeList – we have metadata on the genres of fictional works: for all 22,007 movies in IMDb; for all 2,836 manga in MyAnimeList; for 14,871 literary works out of the 37,815 in Babel; and for 2742 literary works out of 2,911 in ALF, indicating that this information is not systematically included in Wikidata. These metadata allowed us to explore how fictiveness scores vary across different genres. In each dataset, we identified nine main genres (or combinations of genres) to provide a diverse range of themes, with distinct expectations for fictiveness (see Fig. 3C).

This analysis revealed consistent patterns of variation in fictiveness among genres. For example, in IMDb, Babel, and MyAnimeList, fantasy and science fiction have predictably high fictiveness scores. Across Babel and IMDb, fantasy also scores significantly higher than science fiction, likely because it often incorporates supernatural elements that require greater departures from reality compared to the more technologically grounded nature of science fiction. In contrast, historical fiction (i.e., historical fiction in Babel, drama/war in IMDb, and travel writing in ALF) and biographical genres (i.e., diary in ALF, and biography in IMDb and Babel) tend to score very low. Genres like horror, adventure, and romance often display intermediate levels of fictiveness. The use of externally established genre labels to show that fictiveness varies predictably across different types of stories provides an important external validation of the robustness of our metric (see SM for a confirmation that GPT knows the works it annotates).

The lack of statistically significant differences between some genres offers interesting insights as well. In the Babel dataset, historical fiction and tragedy show similarly low levels of fictiveness: works of tragedy frequently involve real characters or those believed to have existed at the time, within real-world settings, aligning them closely with historical narratives – much like Aristotle’s vision of tragedy being grounded in historical figures and events. The lack of statistically significant differences between the epic and the novel in the ALF dataset is also intriguing, particularly considering Bakhtin’s views on the evolution of narrative forms (Bakhtin et al., 2011). According to Bakhtin, the novel is a distinct genre that diverges from the epic by rejecting its representation of an idealized past. However, the fact that their levels of fictiveness are comparable suggests that the difference between the two genres may not lie in the inventiveness of their content. This alignment in fact reflects Bakhtin’s argument that the core distinction between epic and novel ‘is not the factual sources of the epic, not the content of its historical events, nor the declarations of its authors’ (Bakhtin, 1981, p. 16-17).

The MyAnimeList dataset shows that the five genres with the highest fictiveness scores (on the left of Fig. 3C; with fantasy, science fiction, and supernatural) are significantly more fictive overall than the four other genres (on the right; with romance, comedy, drama, and slice of life), while fictiveness does not statistically differ between genres within each group. Statistically significant differences in overall fictiveness are found between all genres in the first cluster and those in the second one (although the plot only shows the significance for the two adjacent genres at the frontier between the two clusters; see SM for full statistics). More specifically, in the less fictive cluster, we observe that characters are consistently invented but plausible (with an average score of 2 and very low variance), while the settings are almost always in the real world (with an average score of 0 and very low variance). Although we removed the MyAnimeList dataset from subsequent analyses due to inconsistent coding patterns, we nevertheless find these preliminary results insightful and in line with our expectations.

### 3.2. Fictiveness is enjoyed by people higher in openness

We then used the IMDb dataset and metadata from Nave et al. (2020), which provided socio-demographic and personality trait data for 3.5 million people and the movies they liked on Facebook. For 690 annotated movies in our dataset, we had the average ‘big five’ personality scores, age, and gender (ranging from 0 to 1, with 0 for men and 1 for women) of the audiences who liked each movie on Facebook. We used linear models with fictiveness as the outcome variable and these characteristics as explanatory variables. The results showed that movies with higher fictiveness scores were enjoyed by audiences who, on average, were significantly higher in openness (ß = 0.02, CI [0.01, 0.04], p < 0.001), lower in conscientiousness (ß = − 0.03, CI [−0.03, − 0.02], p < 0.001), extraversion (ß = − 0.04, CI [−0.05, − 0.03], p < 0.001), agreeableness (ß = − 0.01, CI [−0.02, − 0.003], p < 0.01), and neuroticism (ß = − 0.01, CI [−0.02, − 0.008], p < 0.001). Additionally, these movies were preferred by younger audiences (ß = − 0.20, CI [−0.32, − 0.07], p < 0.01) and were more likely to be enjoyed by males (ß = − 0.02, CI [−0.03, − 0.01], p < 0.001).

**Figure 4.**
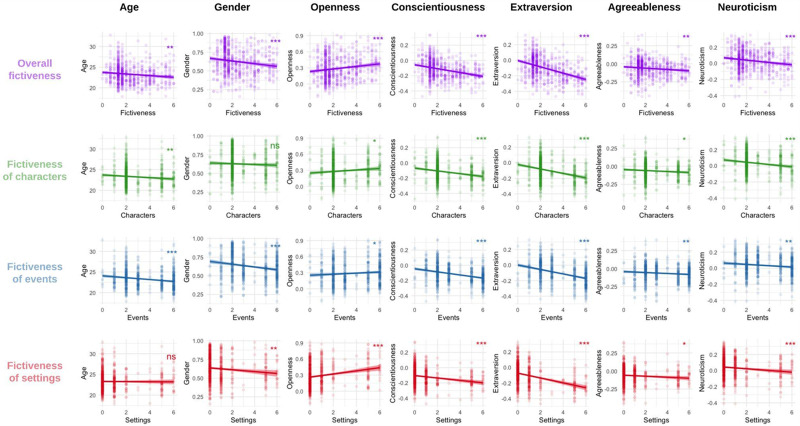
Correlations between personality traits and socio-demographic characteristics of the audiences who ‘liked’ movies on Facebook (N = 3.5 million), in function of the fictiveness of the movies (N = 690 movies).

These results are almost perfectly aligned with previous studies, where we found that people who enjoyed movies with imaginary worlds – indicating high fictiveness of settings – were also higher in openness, younger, and more likely to be male (Dubourg, Thouzeau, Baumard, et al., 2023). Given these parallels, we sought to investigate whether these effects would persist when examining fictiveness at the level of individual dimensions (see Fig. 4). Except for two effects that became non-significant (the effect of gender on the fictiveness of characters and the effect of age on the fictiveness of settings), these findings were replicated across all three dimensions, indicating that the observed relationships between fictiveness and audience characteristics hold for settings, as well as for characters and events.

### 3.3. Fictiveness increases across time

#### Changes in the last century

**Figure 5.**
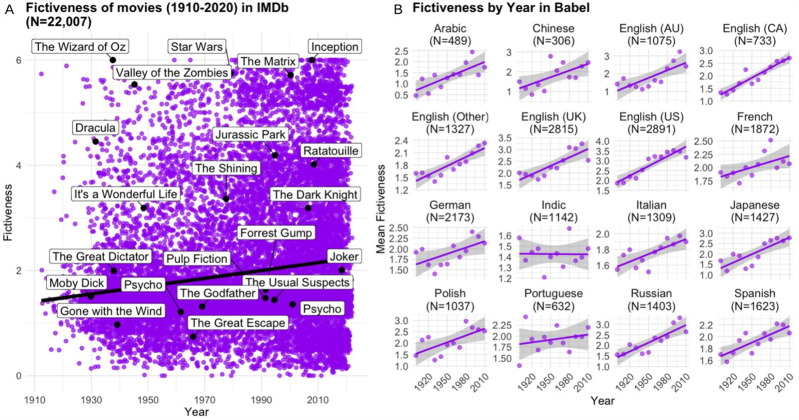
(A) Evolution of fictiveness across time in IMDb. (B) Evolution of fictiveness across time and languages in Babel (with varying y-axis scaling).

To analyse trends in fictiveness over time, we fitted separate linear models for each of our main datasets, focusing on the 20th and early 21st centuries (1900–2021). These independent models allowed us to examine whether the increase in fictiveness was consistent across different narrative forms and media. In Babel, which includes 27,379 literary works published between 1900 and 2021, we observed a significant positive relationship between fictiveness and year (β = 0.0076, CI [0.0071, 0.0081], p < 0.001). In IMDb, based on 22,007 films released between 1900 and 2021, the results were consistent: we found a significant positive effect of year on fictiveness (β = 0.0075, CI [0.0069, 0.0080], p < 0.001; see Fig. 5A). In AMCF, our dataset of 1,240 Chinese literary works from the same period, the increase in fictiveness was even more pronounced (β = 0.0117 (CI [0.0097, 0.0136], p < 0.001). Taken together, these independent analyses reveal a consistent and significant increase in fictiveness across different narrative media and cultural contexts throughout the 20th and early 21st centuries.

In IMDb, the trend persisted in a mixed model with genre as a random effect (ß = 0.0027, CI [0.0023, 0.0031], p < 0.001), indicating the rise was independent of genre. In Babel, a mixed model with language as a random effect confirmed a significant increase of fictiveness across linguistic regions (ß = 0.0096, CI [0.0090, 0.0102], p < 0.001; see Fig. 5B). These findings highlight an overarching shift toward more fictive narratives, robust to variations in genre and language.

We hypothesize that the steady increase in fictiveness we observed reflects a growing audience preference for more fictive stories over time – a demand-driven dynamic where cultural production adapts to meet changing consumer tastes. If this is the case, we should observe not only an increase in the number of fictive stories produced (see previous analysis), but also an increase in their *popularity* relative to less fictive stories.

In our next analysis, we directly test this prediction by examining the relationship between fictiveness and popularity over time. We used IMDb, because we could use the variable of worldwide gross income (‘box-office’) as an indicator of cultural success (available for N = 4,331, from 1925 to 2019). We adjusted all the monetary values (budget and worldwide gross income) for inflation using the U.S. Consumer Price Index by converting all amounts into constant 2020 dollars. We then conducted a series of linear regression analyses, with all variables standardized and gross income and budget log-transformed. The first model (Model 1) includes budget, duration, and year of release as predictors of gross income. This model serves as a baseline, including variables that are known to impact the box office of a given movie. Model 2 extends Model 1 by including fictiveness as an additional predictor to assess its direct effect on box office performance. Finally, Model 3 adds an interaction term between fictiveness and year, allowing us to assess whether the impact of fictiveness on earnings changes over time.

**Figure 6.**
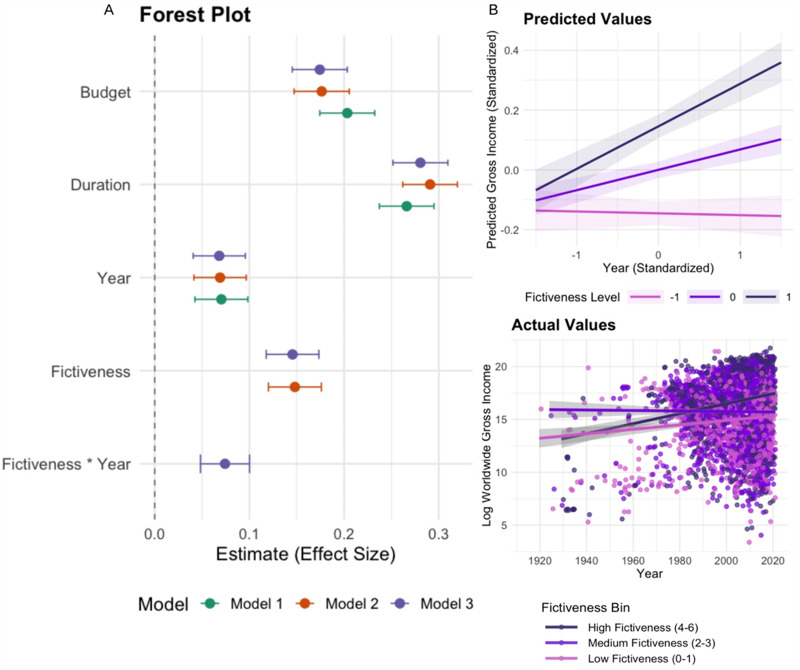
(A) Forest plot of standardized regression coefficients predicting worldwide gross income from budget, duration, year, fictiveness, and the interaction of fictiveness and year. Points represent standardized effect sizes; horizontal lines show 95% confidence intervals. Model 1 includes budget, duration, and year (green); Model 2 adds fictiveness (orange); Model 3 adds the interaction between fictiveness and year (purple). (B) Plot of the interaction effect of year and fictiveness on gross income. Top: predicted values from the regression model. Bottom: actual values, displaying the distribution of log worldwide gross income over time across three bins of fictiveness (with regression lines representing linear model fits between year of release and log worldwide gross income for each bin of fictiveness across time).

In the baseline model (Model 1), budget has a substantial positive effect, with an estimated standardized coefficient of 0.20 (p < .001), indicating that, holding all else constant, movies with higher budgets tend to earn significantly more at the box office. Duration also positively predicts gross income (standardized coefficient = 0.27, p < .001), suggesting that longer films are generally associated with higher earnings. Similarly, year of release shows a positive effect (standardized coefficient = 0.070, p < .001), reflecting the broader increase in box office revenues for more recent films. In Model 2, fictiveness is added as an additional predictor. The estimated coefficient for fictiveness is 0.15 (p < .001), meaning that, all else being equal, movies with higher fictiveness scores tend to achieve higher worldwide gross income. Notably, the coefficients for budget, duration, and year remain significant and positive, with only minor changes in their magnitudes. Additionally, Model 2 shows a lower Akaike information criterion (AIC) (11,538.97) compared to Model 1 (AIC = 11,643.79), indicating a better fit. Model 3 introduces an interaction term between fictiveness and year of release, allowing us to examine whether the impact of fictiveness on box office success has changed over time. In this model, fictiveness continues to show a significant positive effect (standardized coefficient = 0.15, p < .001), and the interaction between fictiveness and year is also positive and significant (standardized coefficient = 0.074, p < .001). This suggests that the association between fictiveness and box office income has strengthened over time: more recent films benefit more from higher levels of fictiveness compared to earlier films in the dataset. Model 3 achieves the best model fit, with the lowest AIC (11,509.53). Overall, these results confirm our hypothesis that fictiveness has become increasingly popular over time, as demonstrated by the growing box-office success of highly fictive films relative to more realistic ones (see Fig. 6).

#### Changes in the very long run

Extending the analysis over the long term, we studied fictiveness trends spanning multiple centuries using literary works from the Babel, ALF, and AMCF datasets, which include works dating back to before 1900.

**Figure 7.**
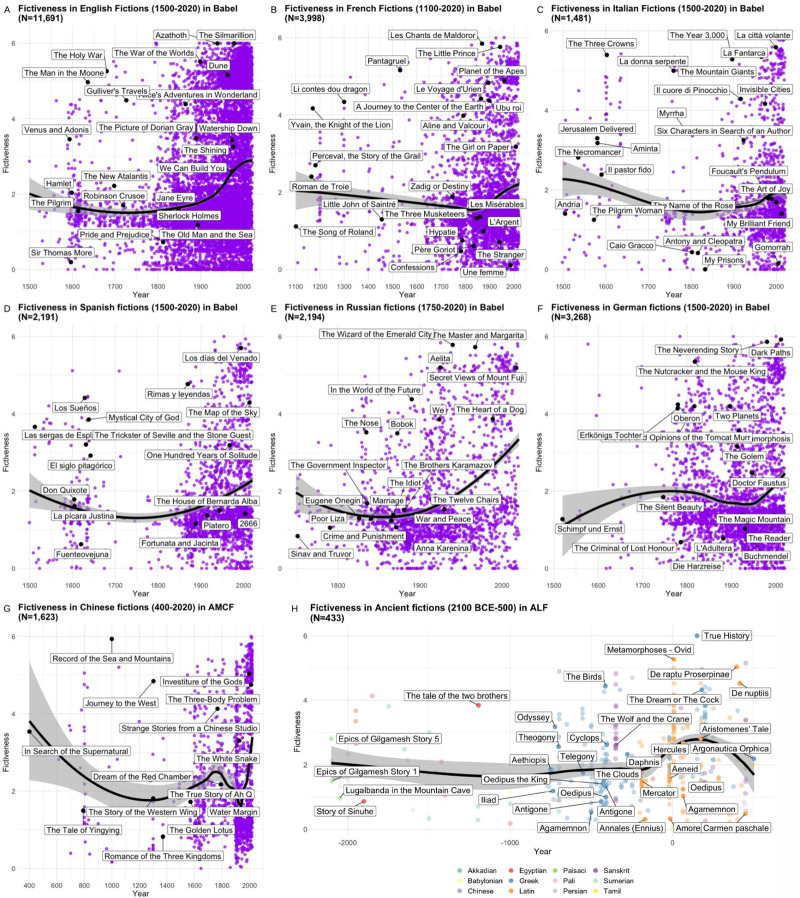
Evolution of fictiveness across linguistic regions. Note that in our analysis, we used a linear model to capture the overall trend of increasing or decreasing fictiveness over time. However, in these graphs, we present a LOESS regression line, which provides a smoother visualization, allowing for the exploration of more fine-grained variations in the data.

The analysis of fictiveness over time in individual languages within the Babel dataset showed a consistent increase across all examined cultures. In English fictions (N = 11,691: β = 0.0049, CI [0.0045, 0.0052], p < 0.001; Figure 7A), French fictions (N = 3,998: β = 0.0009, CI [0.0006, 0.0012], p < 0.001; Figure 7B), Italian fictions (N = 1,481: β = 0.0024, CI [0.0014, 0.0034], p < 0.001; Figure 7C), Spanish fictions (N = 2,191; β = 0.0015, CI [0.0014, 0.0034], p < 0.001; Figure 7D), Russian fictions (N = 2,194: β = 0.0093, CI [0.0083, 0.0103], p < 0.001; Figure 7E), German fictions (N = 3,268: β = 0.001, CI [0.0003, 0.002], p < 0.001; Figure 7F), we found significant positive relationships between fictiveness and year. In Chinese fictions from the AMCF dataset (N = 1,623: β = 0.0003, CI [−0.00001, 0.0007], p = 0.056; Figure 7G), the increase was marginally significant. For ancient fictions, we used the ALF dataset (N = 433) with literary works written between 2100 BCE and 500 CE. The results indicate a significant positive relationship between fictiveness and year (β = 0.00039, CI [0.00019, 0.00058], p < 0.001; Figure 7H).

The statistical models show significant results for a gradual increase in fictiveness over time. As with any historical analysis, there is always a potential concern about sampling biases. However, for these biases to explain the observed trend, they would need to be systematically aligned with time; that is, different kinds of works would have to be preferentially sampled at different periods in a way that mimics a consistent rise in fictiveness. Given the heterogeneity of sources, periods, and languages in our datasets, such a systematic bias is unlikely. This is also why we focused on simple linear models to capture the overall direction of change (while using LOESS regression in the figures to illustrate finer-grained variations). Together, this strategy supports the robustness of the trend we report.

## 4. Discussion

### 4.1. Describing the rise of fiction

We have provided evidence supporting the hypothesis of a steady increase in fictiveness across various countries and time periods, reinforcing the qualitative observations made by scholars in literary theory and history. Our findings indicate a consistent rise in the degree of fictiveness across multiple narrative forms, including Western and non-Western literary works and movies. This trend is observed not only over the course of the 20th and early 21st centuries but also in data spanning from ancient to modern times, suggesting a long-term, gradual shift toward more fictive storytelling.

Consider ancient literature. *The Epics of Gilgamesh* (2100 BCE, Sumeria), *The Tale of the Two Brothers* (1196 BCE, Egypt), and Hesiod’s *Theogony* (700 BCE, Greece) are among the earliest instances of literary works in our dataset (ALF). But these works are actually quite low in fictiveness; they are mostly centred around historical or mythological figures (i.e., believed to have existed) such as Gilgamesh, Œdipus, Agamemnon, or Antigone. The settings, too, are often realistic, grounded in real locations, or locations believed to exist by the people of the time. For instance, *The Epic of Gilgamesh* takes place in recognizable actual cities like Uruk, while *The Odyssey* describes real regions of the Mediterranean, even when venturing to distant islands that were considered part of the real world by contemporary audiences. Such findings are consistent with Aristotle’s view that literature should maintain a connection to history because it heightens the sense of possibility – ‘what did happen is clearly possible, since it would not have happened if it were not’.

Then, we observed in our analysis a peak in fictiveness coinciding with the height of the Roman Empire (27 BCE to 180 CE). Note that qualitative evidence supports the idea that fictiveness serves as a reliable proxy for fictionality here, as some writers in this period intentionally signalled the *fictionality* of their *fictive* stories – and audiences clearly suspended their disbelief in these stories. For instance, Ovid, in *Amores* (16 BCE), explicitly differentiates his poetic creativity from historical reliability, emphasizing that his invented narratives were intended as ‘special lies’. Virgil’s *Aeneid* (19 BCE) provides another example, which later commentators like Macrobius and Bernardus Silvestris understood as a deliberate blending of historical events and inventions. As Green (2002) put it, ‘Macrobius is particularly illuminating about the way in which this *fabula* was received, not simply by himself alone, but by the whole world (*universitas*). Everyone knows this *fabula* to be false, and is aware that it presents only the appearance of truth (*species veritatis*), as if it were true (*pro vero*).’ After this peak, we observe a decline in fictiveness that coincides with the beginning of the decline of the Roman Empire in the third century CE: from this point onwards, stories become less fictive.

In the High Medieval period, we observed highly fictive literary works in France (and likely across the rest of Europe, though data limitations prevented broader modelling), which aligns very well with qualitative observations (Agapītós & Mortensen, 2012). Early texts, such as *Vita Merlini* (circa 1150) and *Roman de Troie* (circa 1155), integrated plausible figures within realistic settings; and *chansons de geste*, such as *The Song of Roland* (circa 1040), featured historical or semi-historical figures. While these narratives were grounded in actual events, they incorporated numerous fictive exaggerations (e.g., prophetic abilities, battles with impossible odds, supernatural interventions in human affairs). In the 1500s, fictiveness was high across various European countries, with a notable peak of fictiveness observed in England, Spain, and Italy during the Renaissance. For instance, *Orlando Furioso* (1516, Italy), by Ariosto, introduced elements like a flying horse and a journey to the moon, and *The New Atlantis* (1627, England), by Francis Bacon, depicted advanced, fictional technologies such as submarines. Some French works from this period were also highly fictive (although the model indicates an overall trend of declining fictiveness in France until the Industrial Revolution); for instance *Les Aventures de Télémaque* (1699) featured interactions with entirely mythical beings like nymphs and gods taking place in fantastical landscapes.

China’s rise in fictiveness began even earlier, with the emergence of *zhiguai xiaoshuo* (sometimes *chih-kuai*; i.e., tales of the anomalies), which flourished during the Tang Dynasty (618–907 CE) but had their roots as far back as the Han Dynasty (although we have no data from this period in our analysis). These stories featured supernatural elements such as giants, dragons, immortals, and ghosts. Such elements are what psychologists today refer to as ‘counterintuitive items’, which violate naturally developing intuitive beliefs about universal ontological domains, like biology or physics, and are, for this reason, appealing to human minds (Boyer & Ramble, 2001; Burdett et al., 2009; Norenzayan et al., 2006; Stubbersfield & Tehrani, 2013). Notable examples include *The Tale of Li Wa* (circa 800 CE) and *The Tale of Miss Ren* (circa 770 CE). While these tales are indeed fictive, whether they are *fictional* remains debated: authors of *zhiguai xiaoshuo* may have considered their craft part of historical storytelling, recounting tales that were circulating rather than explicitly fabricating fiction (Campany, 1996; but see: Dewoskin, 2014). However, given that these narratives include anomalies that could not have been directly observed, one could argue that audiences understood such stories to be distinct from literal truth and did not allow them to influence everyday decision-making.

Moving into the 19th century, literary works became even more fictive after the Industrial Revolution, with novels becoming a dominant form of storytelling. Much like earlier narratives, novels contain invented events, often with embellishments (similar to those in *chansons de geste*, for instance), set against realistic backdrops (similar to most ancient narratives). The distinguishing feature of novels compared to older forms of storytelling, however, lies in the invention of entirely new characters, rather than relying on historical figures. For instance, Daniel Defoe’s *Robinson Crusoe* (1719, England) and Samuel Richardson’s *Pamela* (1740, France) featured invented characters. The 20th and early 21st centuries marked the culmination of this rise, with fictiveness reaching unprecedented levels across all narrative forms, including literature, cinema, and manga. Novels like *The Lord of the Rings* (1954), movies like *Star Wars* (1977), and manga like *One Piece* (1997–present) created highly imaginative worlds filled with fantastical adventures and mythical creatures, showcasing a distinct preference for invented settings and characters (Besson, 2015).

Our analysis of the rise in fictiveness includes two key limitations that, if anything, suggest that the trend we observed may be even more pronounced than the statistical results indicate. The first limitation is a sampling bias. Since our dataset is drawn from user-generated online encyclopedias and platforms, it is likely influenced by modern preferences. Given that contemporary audiences seem to show a greater preference for fictive narratives, it is probable that users have tended to select past works that are more fictive than the broader body of literature from those eras. This means our dataset may overrepresent fictive works from earlier times, thereby underestimating the true rise in fictiveness over time. The second limitation relates to the annotation process using GPT, which might overestimate the fictiveness of ancient works. The model might underestimate the extent to which people historically believed in such stories, leading to an inflated assessment of their fictiveness. Our analysis of the rise in fictiveness may therefore understate the actual trend, due to both a sampling bias favouring more fictive past works and potential overestimation of fictiveness by GPT annotations.

### 4.2. Explaining the rise of fiction

The overall rise in fictiveness over time raises the central question: why has it increased? While further research is needed to rigorously test explanations, we propose five hypotheses to guide future investigations. We propose two main groups of hypotheses: those suggesting a delayed response from producers, and those suggesting shifts in audience preferences over time.

The first group posits that audiences have always preferred more fictive stories, but producers were initially slow to respond. This could be due to two main reasons, leading to two distinct hypotheses: the ‘preference discovery lag’ hypothesis, where producers gradually realized the audience’s true tastes, and the ‘cognitive constraint’ hypothesis, which suggests that people in the past lacked some cognitive tools to create highly fictive stories.

The second group of hypotheses suggests that changes in audience preferences themselves have driven the increase in fictiveness. In this group, we identify three distinct hypotheses. The ‘increase in trust’ hypothesis proposes that early audiences avoided fictive stories due to distrust. Under the ‘decrease in puritanism’ hypothesis, fictiveness was initially frowned upon due to puritanical morality. And the ‘increase of openness’ hypothesis suggests that fictiveness was once seen as uninteresting until an increase in openness and curiosity.

#### The ‘cognitive constraints’ hypothesis

This hypothesis suggests that while audiences may have always preferred fictive stories due to their intrinsic appeal – such as the possibility of exaggerations (Dubourg et al., 2024) – creating highly fictive narratives was cognitively challenging for early storytellers. We argue that this hypothesis is very unlikely for one main reason: the existence of precursors. The very presence of highly fictive stories in the distant past demonstrates that it was *always feasible* to create elaborate fictive content even when broader trends leaned towards more grounded storytelling. Consider examples like *Antheus* (one of the few ancient tragedies featuring entirely invented characters), Homer’s *Odyssey* (with its fantastical islands and mythical creatures), or Aristophanes’ *The Birds* (a story that involved a completely invented society of birds). These works indicate that, even centuries ago, storytellers were capable of creating narratives that were highly fictive. This is in fact visible in our data, which show that many stories from early periods received high fictiveness scores, demonstrating that the cognitive capacity to create such stories existed long before they became prevalent.

#### The ‘preference discovery lag’ hypothesis

This hypothesis posits that producers were initially slow to recognize the audience’s latent preference for highly fictive stories, thus delaying the widespread creation of such narratives. However, we find this explanation unlikely, largely due to the same reason as for the previous hypothesis: the presence of highly fictive precursors in the past – as well as the fact that these precursors were known to other storytellers of both their time and subsequent periods. For example, stories like *The Odyssey* and *The Birds* were well-known, indicating that producers were aware of the level of appeal of more or less fictive content. If they chose to focus on less fictive stories, it is likely due to their observations of what their audience found compelling at the time. Therefore, the existence of these early fictive narratives undermines the argument that audiences always desired such stories, but producers failed to respond. We believe instead that audience preferences have changed over time, and understanding these changes could provide causal explanations of the gradual rise of fictiveness in storytelling. We now turn to the three hypotheses that could explain such changes in audience preferences.

#### The ‘increase in trust’ hypothesis

The ‘increase in trust’ hypothesis suggests that, although people may have always enjoyed the content of fictive stories, they might have been wary of their manipulative potential. The concern is not about the narratives themselves but rather about their perceived capacity to deceive and mislead audiences – much like lies. According to evolutionary psychologists, humans have evolved specialized cognitive mechanisms to detect cheating (i.e., cooperative situations that end up being costly), particularly in social interactions where trust is crucial for cooperation (Cosmides et al., 2010; Sperber et al., 2010). But cheating is not just detected, it is also sanctioned, through the reduced likelihood of future cooperative exchanges with the cheater – whose moral reputation suffers (Altay et al., 2020). According to the evolutionary contractualist theory of morality, sanctioning cheaters is even seen as a moral duty, precisely because it creates a general expectation that cheating will not be tolerated (André et al., 2022).

Telling fictive stories could then be perceived as too close to lying – a type of cheating, where the deceived party ends up bearing the costs. If a story’s fictionality is not made explicit or is ambiguously conveyed, the storyteller risks being seen as a liar rather than a creator of fiction. Historically, influential figures like Plato and Confucius (around the fifth century BCE in Greece and China, respectively) voiced strong disapproval of such narratives, labelling poets and storytellers as ‘liars’ or ‘deceivers’ (Cai, 1999). This deep-seated wariness about the deceptive potential of fictive stories may have suppressed their popularity for centuries. Even today, storytellers may fear their intent being misunderstood. Wolfgang Hildesheimer’s *Marbot: A Biography* (1981) exemplifies this risk (Schaeffer, 1999). The book, which presents Sir Andrew Marbot as a 19th-century British art critic interacting with figures like Goethe and Delacroix, was initially praised as a rediscovery of an overlooked historical figure. Only later did readers learn Marbot was entirely fictive. Hildesheimer claimed, ‘if so many readers and critics fell into the trap of my fabrication, all I can say is that it wasn’t my fault (…) I did not wish to deceive anyone, though I realize now that the revelation of his fictive nature was perhaps too hidden and too weak.’ This illustrates the necessity for clear alignment between the creator’s intent and audience perception, as even unintended ambiguity can lead to perceptions of deceit.

The gradual rise in fictiveness, therefore, may be caused by the increase in trust across societies over time (Martins & Baumard, 2020). As societies became more trustworthy, the fear of deception may have decreased, allowing both creators and audiences to engage more freely with fictive narratives. In trustworthy societies, audiences could increasingly assume that the intent of creators was not to deceive but to entertain. This reassurance also likely extended to the storytellers themselves, who became less apprehensive about being perceived as dishonest or manipulative. But what caused the increase in trust that, according to this hypothesis, allowed the emergence of more fictive narratives? One possible explanation for the rise in trust lies in ecological changes. Increased material security and reduced environmental stress are known to foster trust in others (Martins & Baumard, 2020; Nettle & Saxe, 2022; Vanags et al., 2023). It is plausible that these factors contributed to a societal shift in how fictive stories were perceived. In earlier times, when insecurity and therefore distrust were more prevalent, storytellers may have avoided creating highly fictive narratives to prevent their intentions from being misunderstood as deceitful. With increasing trust, this perceived risk diminishes, opening the way for the emergence of fictive storytelling.

However, we believe this hypothesis is limited in what it can explain. The example of Marbot appears to be an exception rather than the rule – an instance of a literary hoax that retained attention precisely because of its exceptional nature. In general, this hypothesis seems unlikely to fully explain the patterns we observe, given the sophistication of human pragmatic sense, which enables individuals, in general, to carefully distinguish fiction from lies in most cases (Heintz & Scott-Phillips, 2023; Leslie, 1987). Evidence from developmental psychology suggests that even young children are capable of detecting fiction when pragmatic cues are available. For instance, studies show that children as young as two to three years old can differentiate between pretend play and deceit, relying on contextual and communicative signals to interpret the speaker’s intent (Gopnik et al., 1999; Harris, 2000; Rakoczy, 2008; Woolley & Ghossainy, 2013). These findings indicate that humans possess an early-developing and robust capacity to detect fiction, making it implausible that concerns about deception alone could have significantly hindered the emergence of fictive storytelling.

#### The ‘decrease of puritanism’ hypothesis

The ‘decrease of puritanism’ hypothesis suggests that while people may have always appreciated fictive narratives for their intrinsic appeal, they may have been hesitant to consume them due to the risk of moral condemnation. In this view, fictive stories are lumped together with other pleasurable activities condemned by puritanical or ascetic norms, like music, sex, or drug use. Historical contexts such as Neo-Confucian China or Victorian England exemplify this mindset. In these societies, even harmless pleasures, like sexual activity within marriage or masturbation, were often frowned upon. Recent work by Fitouchi et al. (2023) offers an evolutionary rationale for these moral condemnations of harmless pleasures. They argue that puritanical morality may be linked to the belief that resisting temptations of immediate pleasure (i.e., self-control) is necessary for being a reliable and cooperative individual. The ability to delay gratification and suppress self-indulgent desires is viewed as indicative of someone who can be trusted to contribute to collective welfare.

This theory would explain well why fictions are morally condemned: because they are intuitively perceived as self-indulgent sources of pleasure that are themselves perceived as reducing self-control. Qualitative observations lend support to this hypothesis. First, ancient figures like Confucius may not have condemned fictive storytelling (solely) for its potential to mislead, as in the ‘fictive stories as deceptive’ hypothesis, but for distracting people from more virtuous or socially valuable activities. Even in more recent times, the moral condemnation of fiction consumption has been apparent. Accounts from the 19th and early 20th centuries often reveal a condemnation of reading fiction like romances or science fiction novels, which was seen as indulging in mere time-wasting – aligning well with the puritan intuition that indulgence in pleasures impairs self-control. More recently, video games – a modern form of interactive fiction – have often been labelled as addictive or akin to a behavioural disorder, even being included in the DSM-5. Finally, entertainment is sometimes compared to ‘junk food’ by the consumers themselves (Taylor, 2019).

We would therefore expect stories to become more fictive in less puritan societies, where self-indulgent activities are less condemned. Studies on cultural variations in puritanism indicate that large-scale traditional societies tend to be more puritan, whereas small-scale societies and post-industrial, modern ones are generally more permissive (Fitouchi et al., 2023). This shift in attitudes could potentially explain the rise in fictiveness in our analyses. As societies become less puritanical, people can more freely indulge in fiction as a form of entertainment, without moral condemnation. As in the ‘increase in trust’ hypothesis, our results could be linked to variations in trust and, ultimately, in resource prevalence ensuring material security. More secure and affluent environments would lead to a decrease in puritanical attitudes, as self-control becomes less crucial in such environments. This reduction in puritanism, in turn, would create the space for an increase in the fictiveness of stories.

#### The ‘increase in openness’ hypothesis

The ‘increase in openness’ hypothesis, finally, suggests that audiences may not have always enjoyed fictive stories to the same extent that they do today. Unlike the other hypotheses, which posit that external constraints (e.g., storytellers’ cognitive capacities, social trust levels) limited the rise of fictiveness despite an underlying preference for it, this hypothesis implies that audiences in the past simply found fictive stories less interesting. In this view, there was no inherent barrier that kept stories from becoming more fictive – rather, there was a lack of sufficient audience interest in highly fictive content until preferences shifted over time. Another way to see this hypothesis is to consider neophobia – the disinterest or dislike of novel experiences – which is seen as an adaptive response to novel stimuli, which can be dangerous (e.g., toxic food or predators; Mettke-Hofmann et al., 2002; Schaffer et al., 2021).

This hypothesis is supported by evidence linking fictiveness with curiosity for novelty. One study found a positive correlation between enjoyment of fictive settings and levels of exploratory preferences, suggesting that curiosity plays a role in people’s attraction to more fictive content Dubourg, Thouzeau, de Dampierre, et al., 2023), aligning with several other related studies on the relationship between the personality trait of openness to experience and engagement with highly fictive genres (Annalyn et al., 2020; Cantador et al., 2013; Fong et al., 2013; Kraaykamp & Van Eijck, 2005). Therefore, stories that introduced unfamiliar characters, foreign settings, or strange events could have been considered overly novel, making them less engaging for most people in ancient history. ‘[When] lies are made openly, such crude falsity makes no impression on the soul, and gives no pleasure,’ wrote Georges and Madeleine de Scudéry in the preface to their romance *Ibrahim* (1641–1644); ‘how can I be touched by the misfortunes of the Queen of Guindaye, or of the King of Astrobatia, since I know that their kingdoms are nowhere on the universal map, or more precisely, in the realm of things?’ (cited in Paige, 2011).

Why would fictiveness increase, under this hypothesis? Because novelty-based curiosity itself tends to increase in more affluent environments (Dubourg & Baumard, 2024). According to both a life history approach and resource allocation theory, curiosity adaptively varies with environmental factors (Boon-Falleur et al., 2024; Mell et al., 2021; Schiralli et al., 2019). In harsh or unpredictable environments, the risks and opportunity costs associated with exploration are high, making it more advantageous to exploit immediate resources rather than seek information or uncertain future gains. Conversely, in resource-rich environments, individuals can afford short-term costs for potential long-term benefits, which encourages greater curiosity and exploratory behaviour (Frankenhuis & Gopnik, 2023; Jacquet et al., 2019). Research across multiple species supports this view, showing that individuals in more affluent conditions tend to exhibit more exploratory behaviours (Forss et al., 2015; Katz & Naug, 2015; Mettke-Hofmann et al., 2002; Sharpe et al., 2002; van Schaik et al., 2016). This has been observed in humans too. For example, children from families with higher socio-economic status show greater levels of curiosity and creativity (Menardo et al., 2017; Oh et al., 2022; Shah et al., 2023; Xu et al., 2023; Zhang et al., 2018), and societies with higher GDP levels tend to have higher openness (Inglehart, 2020; Korotayev et al., 2019). These findings suggest that increased material security and reduced environmental stress lead to higher levels of curiosity, potentially contributing to the growing popularity of highly fictive narratives over time – which are, by definition, more novel to the audience than less fictive stories.

### 4.3. Concluding remarks

In this paper, we have observed a trend toward more fictive stories and explored hypotheses to explain why such narratives are increasingly accepted by consumers. For audiences to enjoy fiction, they must trust their ability to distinguish fiction from reality, feel secure that engaging with fiction won’t be morally condemned as indulgent, and find novel content appealing.

These changes in people’s psychology could explain why fictive stories are more accepted. But they do not yet address why such stories hold intrinsic appeal. What is the added value of fictive stories? We propose that fictiveness is not inherently appealing for its own sake but because it enables the crafting of exaggerated content that heightens emotional responses from audiences (Dubourg & Baumard, 2022a; Dubourg et al., 2024; Nettle, 2005). This phenomenon is analogous to the concept of superstimuli observed in the animal world (Barrett, 2010). Superstimuli are artificially amplified versions of natural stimuli that elicit stronger reactions than the original. For instance, in herring gulls, artificial eggs bigger than natural eggs elicit enhanced nesting behaviour in female adults. In chicks, dummy models of parents with more contrasted colours on their bills elicit enhanced pecking behaviour (Tinbergen, 1953b). In stickleback fish, male adults prefer to fight dummy models with brighter red than real male adults and prefer to escort dummy round-bellied models rather than real egg-bearing females (Tinbergen, 1953a). Similarly, in humans, superstimuli like the exaggerated features of fictional characters (e.g., superheroes’ impossible strength; Burch & Widman, 2023), settings (e.g., imaginary worlds; Dubourg & Baumard, 2022b), or monsters (e.g., large size and sharp teeth and claws; Clasen, 2012; Morin & Sobchuk, 2023) amplify our cognitive and emotional responses (see Dubourg et al., 2024, for a review). Fictive stories serve as powerful entertainment technology precisely because they enable storytellers to exaggerate elements beyond what exists in reality, creating narratives that are more gripping. By tapping into our psychological preferences, fictive stories can amplify the psychological rewards of engaging with them. This makes fictiveness a means to an end: the creation of stories that better entertain us.

## Supporting information

Dubourg et al. supplementary material Dubourg et al. supplementary material

## Data Availability

Data and scripts are all available on OSF: https://osf.io/kdtzf/.

## Supplementary material

The supplementary material for this article can be found at https://doi.org/10.1017/ehs.2025.10011.

## Research transparency and reproducibility interest

The annotated datasets and the annotation and analysis script are all available on OSF (https://osf.io/kdtzf/).
