# Supplementary material for: Quantifying and explaining the rise of fiction
Source: Evol Hum Sci. 2025 Jul 14;7:e20. doi: 10.1017/ehs.2025.10011 (PMC12344597; doi:10.1017/ehs.2025.10011)
Supplement: Dubourg et al. supplementary material [file S2513843X2510011Xsup001.docx]

**SUPPLEMENTARY MATERIALS**

**Quantifying and explaining the rise of fiction**

Edgar Dubourg^1 a^, Valentin Thouzeau^1^, Nicolas Baumard^1^

^1^ Institut Jean Nicod (Paris), ENS, EHESS, CNRS, PSL

^a^ Corresponding author: edgar.dubourg@gmail.com

[1. Datasets 2](#_Toc192942368)

[1.1. Summary 2](#_Toc192942369)

[1.2. Babel 2](#_Toc192942370)

[2. Method 4](#_Toc192942371)

[2.1. Scale 4](#_Toc192942372)

[2.2. Prompt 5](#_Toc192942373)

[3. Descriptive statistics 6](#_Toc192942374)

[3. Validity checks 9](#_Toc192942375)

[3.1. Convergence with another LLM 9](#_Toc192942376)

[3.2. GPT’s capability of retrieving the date of literary works 11](#_Toc192942377)

[3.3. Comparisons between genres 13](#_Toc192942378)

# 1. Datasets

## 1.1. Summary

The datasets analyzed in this study represent a variety of fictional works across different media, time periods, and cultural contexts. **Table S1** summarizes the key characteristics of the datasets, including their sources, the number of works, and temporal coverage. **Figure S1** presents the number of works across years for each dataset.

| **Dataset** | **Source** | **Number of works** | **Date of first work** | **Date of last work** | **Reference** |
| --- | --- | --- | --- | --- | --- |
| IMDb | IMDb.com | 22,007 | 1914 | 2019 | / |
| Babel | Wikidata | 37,815 | -800 | 2019 | / |
| AMCF | Baidu + Manual extraction | 1,752 | 400 | 2019 | Zhong et al., 2024 |
| ALF | Wikipedia + Manual extraction | 2,911 | -2100 | 1800 | Baumard et al., 2020 |
| MyAnimeList | MyAnimeList.com | 2,836 | 1931 | 2019 | / |

**Table S1. Description of each dataset.**


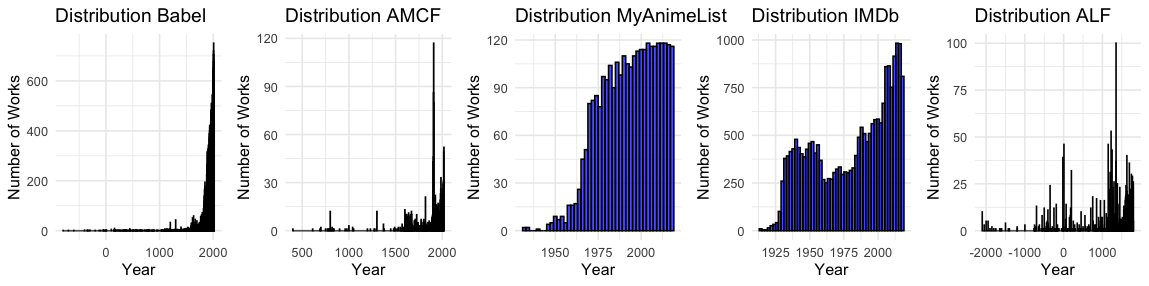


**Figure S1. Distribution of works across time in each dataset.**

## 1.2. Babel

Babel is a dataset of 45,110 literary works extracted from Wikidata (37,815 being retained for analysis in a subsequent step). Because we built this dataset for this study, we present here the extraction methodology.

We rely on an extracted list of works through the property (P31) of the instance variable of Wikidata named “literary work” (Q7725634) in the Wikidata Query Service (N = 246,514 literary works). Each work is associated with a unique identifier (Wikidata ID). For each Wikidata ID, our algorithm constructs a SPARQL query. The query is designed to fetch specific pieces of information about each literary work.

For each literary work in this dataset, we aim to gather central variables, essential for studying the evolution of literary texts: the name of the text (Title), the year of release (Date), the type of text (Type), the name of the author (Author), the language of work (Language), the country of release (Country), and the genres (Genre). Specifically, here are the multiple fields retrieved from the Wikidata IDs: subject, instance, author, publication date, language of writing, genre, country of origin, and a link to the corresponding Wikipedia page. At the end of the extraction, we had 246,514 unique titles.

With the combination of publication date and inception date, we retrieved 111,505 year labels (54.77% Missing Data). With the addition of the author’s year of birth, we additionally retrieved 70,907 labels. For each title’s Year, we therefore use publication date or inception date, if not available the author’s year of birth to which we add 35 years, to approximate a date where the authors were more likely to have written the text. Therefore, we totalized 182,412 year labels out of the 246,514 literary works, with a remaining 64,102 years Missing Data (26 %). With the field country of origin, we retrieved 90,764 country labels (63.18% missing data). With the addition of author’s country of citizenship and author’s country of birth metadata, we additionally retrieved 103,240 new labels. For each title’s Country, we therefore use country of origin, if not available author’s country of citizenship, and if not available author’s country of birth, in this order. Therefore, we totalized 193,975 country labels out of the 246,514 literary works, with a remaining 52,510 countries missing data (21.30%). For the field language, we retrieved 190,281 language labels (22.81% missing data).

We encountered a challenge with the multiplicity of related labels in the metadata due to Wikidata’s diverse annotations. For instance, entries like “Alsace,” “Paris,” “Strasbourg,” and “French Third Republic” were all related but inconsistently labeled under the field for country. Similarly, the language field contained variations like “Middle High German,” “Deutsch,” and “Swiss German,” which all refer to German. To address this, we harmonized both columns through iterative assessments, grouping related labels into broader categories. To further simplify linguistic representation, we created a new column for linguistic regions. This column prioritized the harmonized language if available and otherwise inferred the language based on the country specified. This ensured consistent grouping of works into broader linguistic regions. We then removed every row with a missing value in any of the fields for title, year or language.

We proceeded with several cleaning steps to refine the dataset. First, we removed identifiable poets because they were disproportionately prolific, such as Su Shi and Charles Baudelaire. Additionally, we addressed the issue of Rabindranath Tagore, who had 1,838 works listed in Wikidata, likely due to separate entries for individual poems or short stories. To prevent this from artificially inflating the dataset, we retained only works associated with a Wikipedia page, ensuring a more balanced representation. We also removed languages with fewer than 100 works, ensuring that only languages with sufficient representation were retained for meaningful modeling and analysis (number of languages: . Finally, to reduce the dataset size while maintaining representativity, we implemented a sampling step. We limited the dataset to a maximum of 30 randomly selected works per year per language. For example, if there were 60 French works from 1980, we randomly retained 30. This step primarily reduced the overrepresentation of works from the second half of the 20th century and the 21st century, resulting in a less skewed distribution while preserving diversity.

We ended up with the 45,110 works in the Babel dataset, which is a list of titles from 35 different languages (see **Table 2**).

| **Language** | **N** | **Language** | **N** |
| --- | --- | --- | --- |
| English (UK) | 5295 | Slovene | 596 |
| French | 5292 | Chinese | 536 |
| German | 4211 | Hebrew | 512 |
| English (United States) | 3841 | Turkish | 447 |
| Spanish | 2682 | Finnish | 439 |
| Russian | 2469 | Greek | 425 |
| Indic | 2053 | Serbian | 424 |
| Italian | 2005 | Danish | 420 |
| English (Other) | 1810 | Hungarian | 370 |
| Japanese | 1790 | Norwegian | 351 |
| Polish | 1489 | Indonesian | 304 |
| English (Australia) | 1199 | Persian | 230 |
| Portuguese | 1115 | Latin | 211 |
| Swedish | 993 | Romanian | 194 |
| English (Canada) | 843 | Korean | 191 |
| Arabic | 750 | Armenian | 130 |
| Dutch | 715 | Albanian | 104 |
| Czech | 674 |  |  |

**Table 2**. **Number of literary works per language in Babel.**

# 2. Method

## 2.1. Scale

To systematically assess fictiveness, we developed three distinct scales for characters, events, and settings. Each scale quantifies the extent of deviation from real-world plausibility, as defined by the standards of the time and place. **Table 3** presents the detailed criteria for each level across the three referents, enabling consistent and systematic annotation.

| **Referent** | **Scale** |
| --- | --- |
| Events | **0**: Actual historical events, or believed to have occurred, by the standards of the time and place.  **1**: Actual historical events with invented details, by the standards of the time and place.  **2**: Invented historical events whose occurrence would be possible, by the standards of the time and place.  **3**: Invented historical events whose occurrence is not probable, by the standards of the time and place.  **4**: Invented historical events whose occurrence is not possible but involves minor violations of real-world laws, by the standards of the time and place.  **5**: Invented historical events whose occurrence is not possible but involves moderate violations of real-world laws, by the standards of the time and place.  **6**: Invented historical events whose occurrence is not possible and requires extreme violations of real-world laws, by the standards of the time and place. |
| Characters | **0**: Real characters, or believed to have existed, by the standards of the time and place.  **1**: Real characters with invented biographical details, by the standards of the time and place.  **2**: Invented characters whose existence would be possible, by the standards of the time and place.  **3**: Invented characters whose existence is not probable, by the standards of the time and place.  **4**: Invented characters whose existence is not possible but involves minor violations of real-world laws, by the standards of the time and place.  **5**: Invented characters whose existence is not possible but involves moderate violations of real-world laws, by the standards of the time and place.  **6**: Invented characters whose existence is not possible and requires extreme violations of real-world laws, by the standards of the time and place. |
| Settings | **0**: Real geographical locations, or believed to exist, by the standards of the time and place.  **1**: Real geographical locations with invented details, by the standards of the time and place.  **2**: Invented locations whose existence would be possible, by the standards of the time and place.  **3**: Invented locations whose existence is not probable, by the standards of the time and place.  **4**: Invented locations whose existence is not possible but involves minor violations of real-world laws, by the standards of the time and place.  **5**: Invented locations whose existence is not possible but involves moderate violations of real-world laws, by the standards of the time and place.  **6**: Invented locations whose existence is not possible and requires extreme violations of real-world laws, by the standards of the time and place. |

**Table 3. Fictiveness scales for characters, events and settings**.

## 2.2. Prompt

Our prompt starts by specifying the referent, directing the LLM to focus on a particular aspect of the narrative—protagonists, events, or settings; then it asks the LLM to evaluate the fictiveness of a given title with the specified scale, and to provide a brief explanation of the evaluation. Guidance is provided on how to handle works that may not be within the LLM’s dataset, asking the model to assign “NA” (for *Not Applicable*) for unfamiliar texts. Here is the full prompt used to guide LLMs in this annotation process:

“Evaluate the literary work based on the specified referent using the following scale. *[Insert Scale]*. You must assess the degree of invention, probability, and possibility according to the worldview and beliefs of people in the historical period of the work, without applying modern standards for ancient works. You must evaluate only *[the main protagonists/the events/the settings]* and ignore any other elements of the work. Provide a brief explanation, and conclude the explanation with the score formatted as Score= followed by the numerical value, with no text or symbol after the score. If you are unfamiliar with the work, assign the score as NA. The work is: *[Insert title]* by *[Insert author]* written in *[Insert date]*.”

The instruction to provide a brief explanation followed by a score formatted as “Score=” and the numerical value ensures easy and systematic extraction of scores. This standardized format facilitates efficient compilation of annotations and minimizes ambiguity. The instruction to assign a score of “NA” when unfamiliar with the work addresses the risk of hallucination by large language models, which might otherwise generate speculative or inaccurate information.

We then systematically removed works from each dataset if at least one of the three scores (events, characters, or settings) was marked as NA. This process led to the removal of 25 works from ALF (remaining N = 2,911), 266 works from AMCF (remaining N = 1,752), 6,748 works from Babel (remaining N = 37,815), 2,529 movies from IMDb (remaining N = 22,007), and 525 entries from MyAnimeList (remaining N = 2,836). The GPT justifications for assigning a score of NA demonstrate this added part was crucial, as GPT often begins by returning “I am not familiar with” or “I’m not familiar with,” followed by the title of the work. For example, in the IMDb dataset, among the 2,529 movies removed due to missing annotations, 1,962 of the settings annotations began with this standardized phrasing.

For Babel, the inclusion of a diverse range of works from Wikidata posed unique challenges, particularly with non-narrative forms like poetry. Poetry often lacks the clear narrative structure required for consistent annotation on our scales, making it ambiguous to assess using the criteria developed for events, characters, and settings. To address this, we added a specific instruction in the prompt for the Babel dataset: “If the work is non-narrative (like poetry), assign the score as NA.” This ensured that annotations remained consistent and avoided forcing inappropriate evaluations. Among the 6,748 works removed from Babel, more than 1,000 appear to have been excluded due to their non-narrative format, such as poetry. To identify this, we analyzed the GPT justifications and counted instances where the term “non-narrative” was explicitly mentioned in such output of excluded works, reflecting the phrasing from the prompt likely leading to the NA score. For example, in the “events” annotation column, 1,257 outputs (out of the 6,748 excluded works) included this specific term.

# 3. Descriptive statistics

**Table 4** presents the summary statistics for fictiveness scores across characters, events, settings, and overall fictiveness for each dataset. **Figure 2** complements these statistics by illustrating correlations between scores within each dataset, offering insights into their relationships and distributions.

| **Dataset** | **Mean Fictiveness of characters** | **SD Fictiveness of characters** | **Mean Fictivenes of events** | **SD Fictiveness of events** | **Mean Fictiveness of Settings** | **SD Fictiveness of settings** | **Mean Overall fictiveness** | **SD**  **Overall fictiveness** |
| --- | --- | --- | --- | --- | --- | --- | --- | --- |
| IMDb | 2.33 | 1.18 | 2.90 | 1.61 | 0.58 | 1.33 | 1.92 | 1.17 |
| Babel | 2.26 | 1.34 | 2.52 | 1.73 | 1.11 | 1.72 | 2 | 1.43 |
| AMCF | 2.53 | 1.45 | 2.78 | 1.70 | 1.51 | 1.79 | 2.28 | 1.46 |
| ALF | 2.29 | 1.36 | 3.01 | 1.42 | 1.08 | 1.51 | 2.13 | 1.20 |
| MyAnimeList | 3.47 | 1.66 | 4.12 | 2 | 2 | 2.24 | 3.2 | 1.7 |

**Table 4. Summary statistics of fictiveness scores for each referent and dataset.**


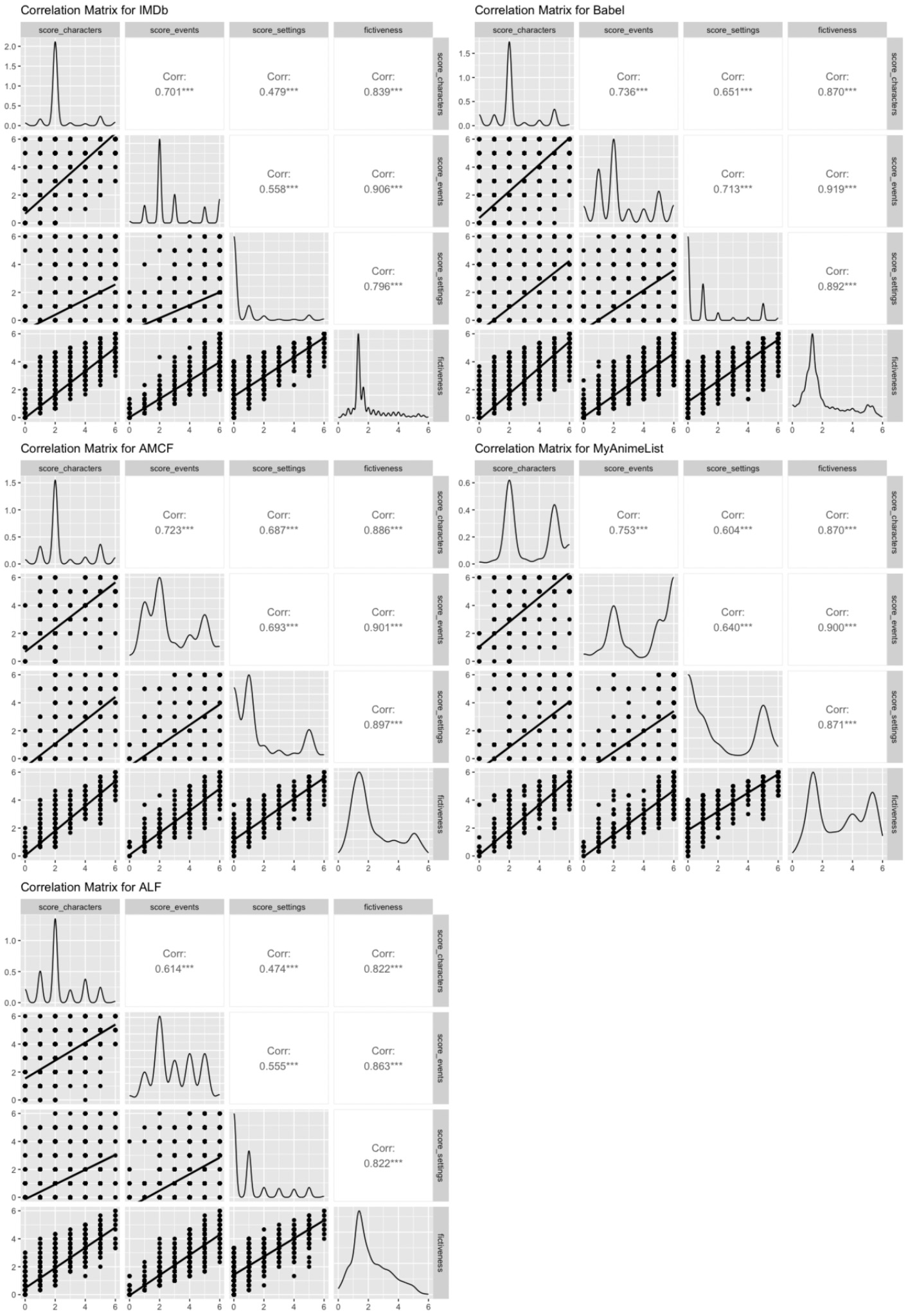


**Figure 2. Correlation Matrices for Scores Across Datasets.** This figure displays correlation matrices for the four scores (characters, events, settings, and fictiveness, which is the average of all three scores for each work) across all datasets. Each matrix includes scatterplots (bottom-left), density distributions (diagonal), and correlation coefficients (top-right).

To ensure the robustness of our analyses, we examined the proportion and distribution of works that were excluded due to missing annotations (i.e., instances where GPT returned “NA” because it lacked sufficient familiarity with a work). Across our five datasets, the proportion of deleted cases remained relatively low: 14.96% in Babel (6,748 out of 45,110), 8.16% in AMCF (266 out of 3,258), 14.84% in MyAnimeList (525 out of 3,537), 10.31% in IMDb (2,529 out of 24,541), and only 0.85% in ALF (25 out of 2,936). In addition to examining percentages, we compared the temporal distribution of excluded (“Deleted”) and retained (“Included”) works within each dataset. Density plots (**Figure 3**) show that the distributions of deleted and included works follow similar temporal patterns, with no clear evidence of systematic bias that would disproportionately affect certain periods. While some variations exist—particularly in MyAnimeList—the deleted works are generally spread across the same time periods as the included ones. This suggests that the exclusion of these cases is unlikely to bias our findings regarding temporal trends in fictiveness.


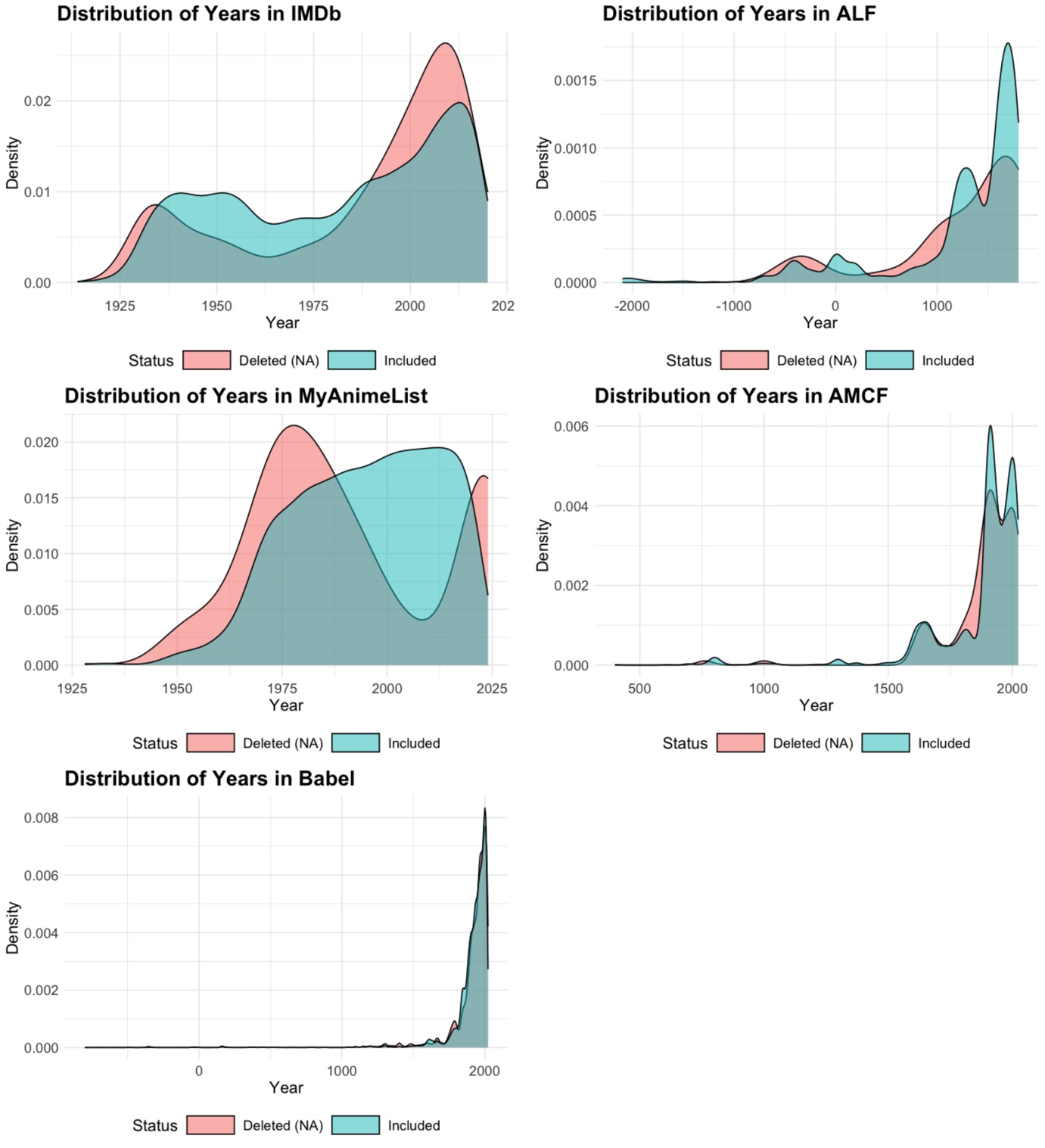


**Figure 3.** Density plots comparing the temporal distribution of included and deleted (NA) works across the five datasets.

# 3. Validity checks

## 3.1. Convergence with another LLM

To validate the reliability of GPT’s annotations, we assessed their convergence with those generated by another large language model, DeepSeek. This model was chosen for its comparable scale and extensive training data, ensuring that it possessed sufficient background knowledge to perform the task. Using identical prompts and annotation scales, we re-annotated a randomly selected set of 100 works of fiction, drawn equally from five different datasets. Each work was evaluated along three key dimensions—characters, settings, and events—before systematically comparing the outputs of the two models.

Replicating results obtained with GPT using DeepSeek is interesting because the two models differ in both training data and underlying architecture, offering a way to test the robustness and generalizability of findings. GPT models, such as OpenAI’s, are trained primarily on a mix of publicly available and licensed data, whereas DeepSeek is specifically trained on a dataset with a significant focus on Chinese-language and Asian cultural contexts. Additionally, their architectures and optimization processes may vary, influencing how they process and generate text. If similar results emerge from both models, it suggests that the findings are not artifacts of a specific training corpus or model bias but rather reflect deeper patterns in the data or the problem being studied. Conversely, discrepancies between the models can reveal the impact of training data, tokenization differences, or architectural design on the observed outcomes, helping refine methodologies and interpretations in computational research.

To assess the alignment between the two models, we first computed the intraclass correlation coefficient (ICC), selecting a two-way random-effects model with absolute agreement (ICC(A,1)), which accounts for systematic differences between annotators. The resulting ICC indicated a moderate level of agreement (ICC = 0.647, 95% CI [0.537, 0.729], p < 0.001), suggesting that while the two models produced similar annotations, they were not perfectly aligned. Agreement varied across datasets. The highest convergence was observed for AMCF (ICC = 0.79) and Babel (ICC = 0.74), followed by IMDb (ICC = 0.70), while lower agreement was found for ACF (ICC = 0.56) and MyAnimeList (ICC = 0.41). These differences likely reflect variations in the nature of the works within each dataset, as well as potential biases in how each model processes different genres or media formats. Despite these discrepancies, perfect agreement was not a requirement for this study, as our primary goal was to ensure that the two models captured similar relative patterns rather than producing identical absolute scores.

Since our goal is to analyze historical trends rather than rely on exact categorical labels, proportional relationships between annotations were more relevant than absolute equivalence. To confirm that the two models captured the same underlying structure in the data, we computed Pearson and Spearman correlation coefficients, which measure linear and monotonic associations, respectively. The results indicated a strong positive correlation (Pearson’s r = 0.681, Spearman’s ρ = 0.696), showing that while the absolute scores differed, the relative ranking of works was largely preserved. Agreement varied across datasets, with the highest correlations observed for AMCF (r = 0.813, ρ = 0.819) and IMDb (r = 0.750, ρ = 0.787), followed by Babel (r = 0.740, ρ = 0.700) and ALF (r = 0.651, ρ = 0.671), while MyAnimeList showed the weakest alignment (r = 0.464, ρ = 0.452).

To further account for variability across different fiction types and annotation dimensions, we implemented a linear mixed-effects model predicting GPT scores from DeepSeek scores while including dataset and dimension as random effects. This approach controlled for systematic differences in annotation tendencies across datasets and for variations between dimensions. The model confirmed a strong predictive relationship between the two models (β = 0.672, SE = 0.059, t = 11.477, p < 0.001), indicating that DeepSeek scores reliably predicted GPT scores but with a slight systematic difference, as shown by the positive intercept (β = 1.178, SE = 0.395, p = 0.042). The random-effects structure revealed that annotation differences were most pronounced at the dimension level (σ² = 0.301), followed by variation across individual works (σ² = 0.200) and then datasets (σ² = 0.165). This pattern is reassuring, as greater variation across dimensions and individual works aligns with expectations. The lower variation across datasets is consistent with our objective of comparing them, suggesting that despite differences in media formats, the overall scoring framework remains stable across sources. These results indicate that while GPT and DeepSeek differ in absolute scoring, they remain strongly aligned in relative terms.

## 3.2. GPT’s capability of retrieving the date of literary works

Before deploying GPT to annotate all works, it was crucial to check the validity of its annotations in terms of accuracy. This necessity arises from the inherent black box nature of GPT-4; the specific data it has been trained on is not fully transparent, necessitating rigorous validation to ensure that the LLM we use “know” the works we want to annotate.

We used the ability of GPT to accurately date the works as an initial indication of its familiarity with them. If GPT could correctly assign a date to a work, it provided a preliminary indication that the model had at least some knowledge about the work. While this approach cannot directly verify GPT’s understanding of the work’s content, it served as a first step in assessing the model’s familiarity with the dataset and its potential reliability in providing annotations.

We subsampled a portion of the dataset. Given that our dataset is highly skewed, with a predominance of entries from the 20th century, we cannot rely on a simple random sample as it would disproportionately represent recent works. To ensure a comprehensive evaluation of GPT’s capacity to accurately annotate metadata for both recent and ancient works, we employ a stratified random sampling method. This involves randomly selecting up to 10 works from each century, or including all works if fewer than 10 exist for that century (N = 266). This approach allows us to create a balanced sample that accurately reflects the temporal diversity of our dataset.

Out of the 266 sampled literary works, GPT annotated 237 works, indicating it had no information on 29 works (10.9%). We considered an absolute difference of 5 years between the dates provided by GPT-4 and those in Wikidata (our benchmark) as successful annotations. Under this criterion, GPT-4 correctly annotated a third of the literary works. While it made errors on the remaining two-thirds, the discrepancies were not substantial, with an average absolute difference of 50 years (Figure **4.A. and B.**). Importantly, there was no discernible pattern of inaccuracy; GPT-4 did not consistently overestimate or underestimate dates, nor did it show a bias toward certain periods. When we aggregated the data at the level of centuries, the accuracy of GPT-4, using Wikidata as the benchmark, was remarkably high at 96.6% (**Figure 4.C.**).

We then realized that GPT might, in some cases, outperform our benchmark, Wikidata, because it synthesizes information from a broader range of sources. Unlike Wikidata, which relies on the accuracy of individual contributions, GPT could benefit from a “wisdom of the crowd” effect, where aggregated responses are often more accurate than those of a single individual. To test this hypothesis, we manually verified (using online sources) the 134 discrepancies between GPT’s annotations and those in Wikidata.

Our manual checks revealed that GPT was correct in 36.6% of these cases, compared to 33.6% for Wikidata (**Figure 4.D.**). For 20% of the works, both GPT and Wikidata appeared plausible, as their annotations fell within the confidence intervals reported by sources— due to the uncertainty surrounding ancient works. In 9% of cases, both GPT and Wikidata provided incorrect dates. This means that more than half of the time when GPT’s annotations diverged from Wikidata, it was actually correct.

This observation underscores the importance of carefully selecting benchmarks when evaluating the accuracy of large language models. In certain information extraction tasks, LLMs like GPT may exceed the reliability of traditional benchmarks.


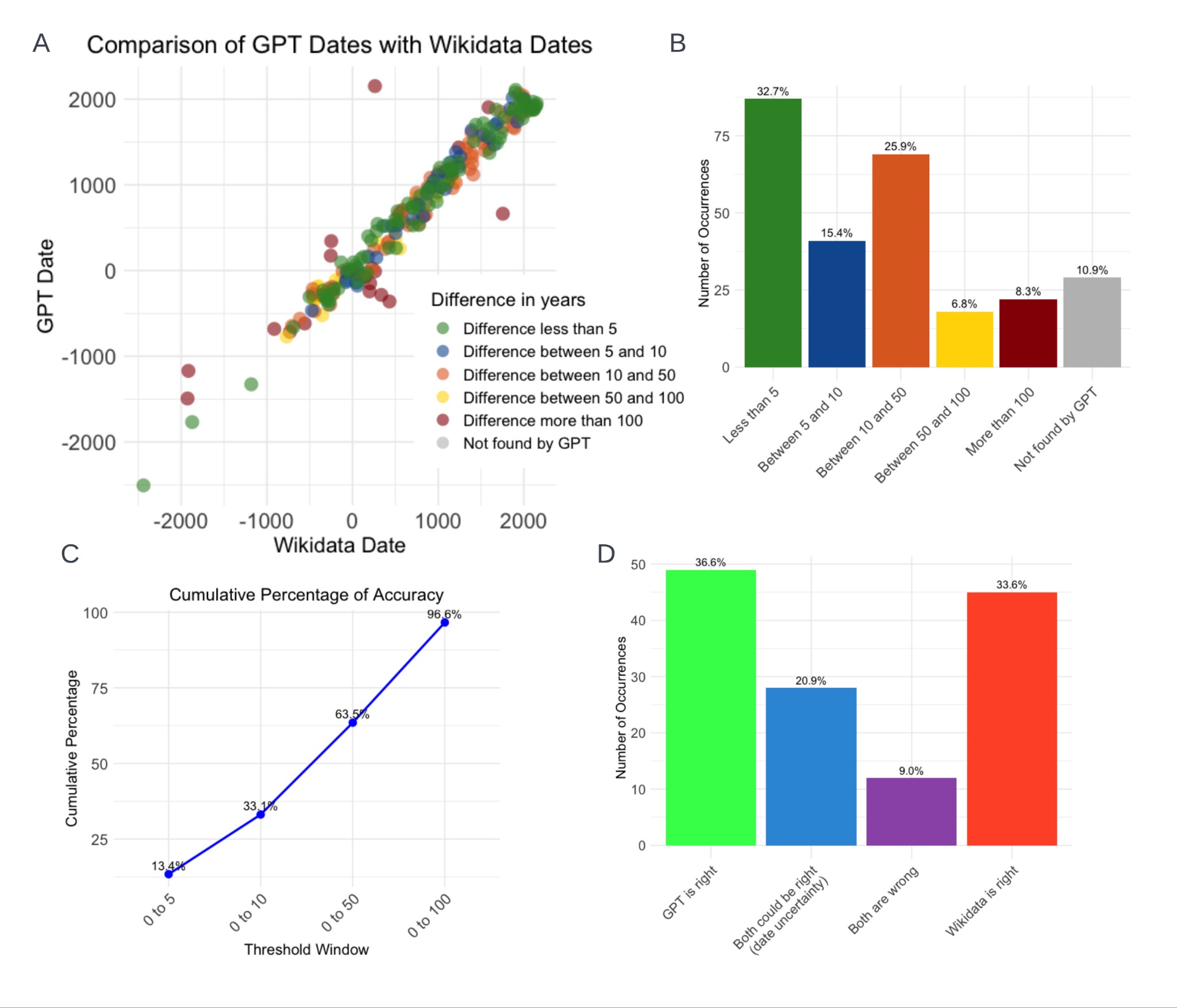


**Figure 4. A.** Comparison of dates provided by GPT with those from Wikidata. **B.** Distribution of errors categorized by error range (e.g., “Less than 5” indicates that 32.7% of GPT’s outputs deviated by fewer than 5 years from the Wikidata date). **C.** Cumulative accuracy percentage across increasing error thresholds. **D.** Proportion of accurate annotations after manual validation of discrepancies greater than 5 years.

## 3.3. Comparisons between genres

To explore how fictiveness scores vary across genres, we conducted statistical comparisons of genres in four datasets: IMDb, Babel, ALF, and MyAnimeList. For each dataset, we focused on nine main genres or genre combinations, ensuring a diverse range of themes. Full statistical details of these comparisons are presented in **Figure 5 to 8**.

**
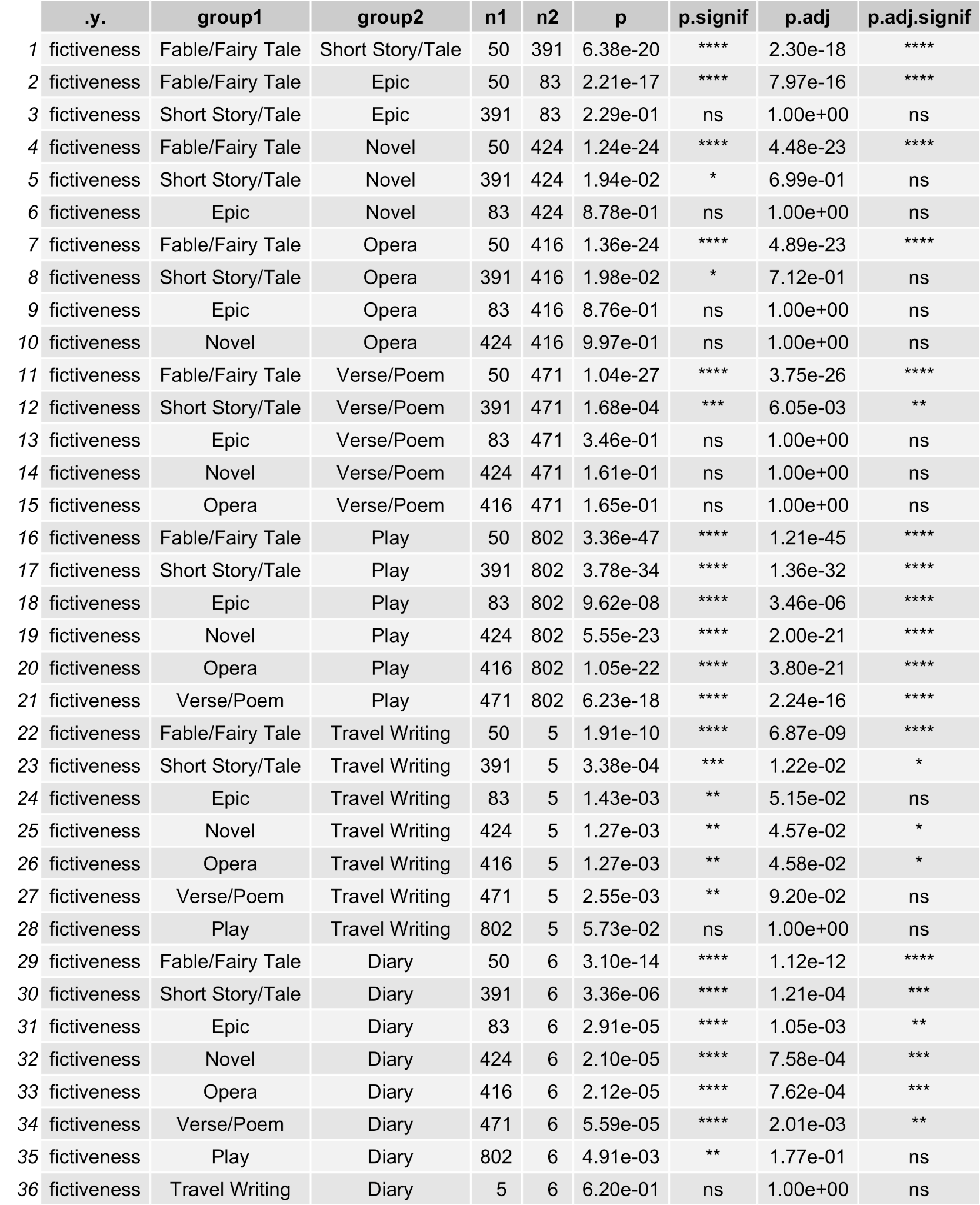
Figure 5. Comparisons of fictiveness between the 9 main genres in ALF.**

**
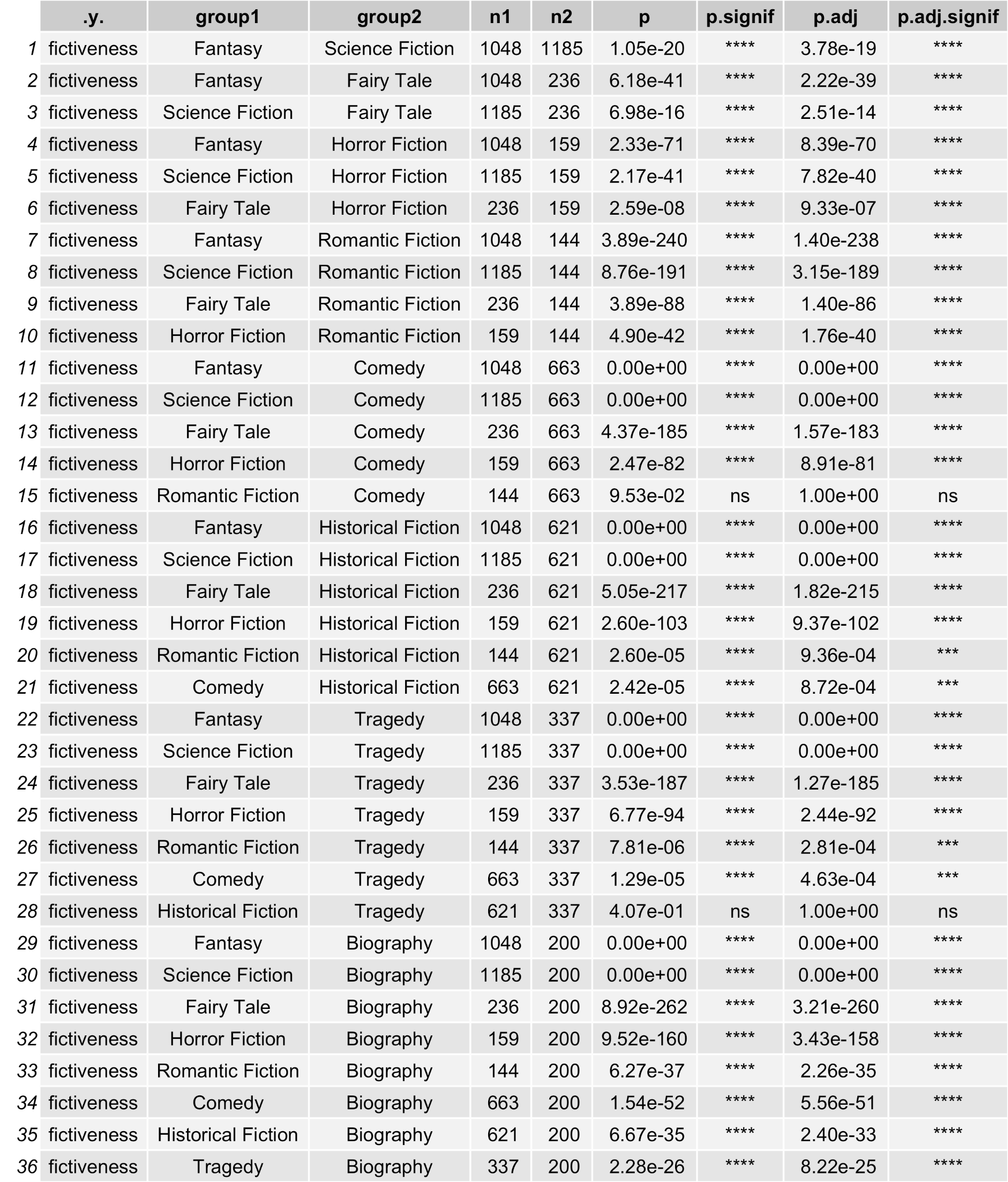
Figure 6. Comparisons of fictiveness between the 9 main genres in Babel.**

**
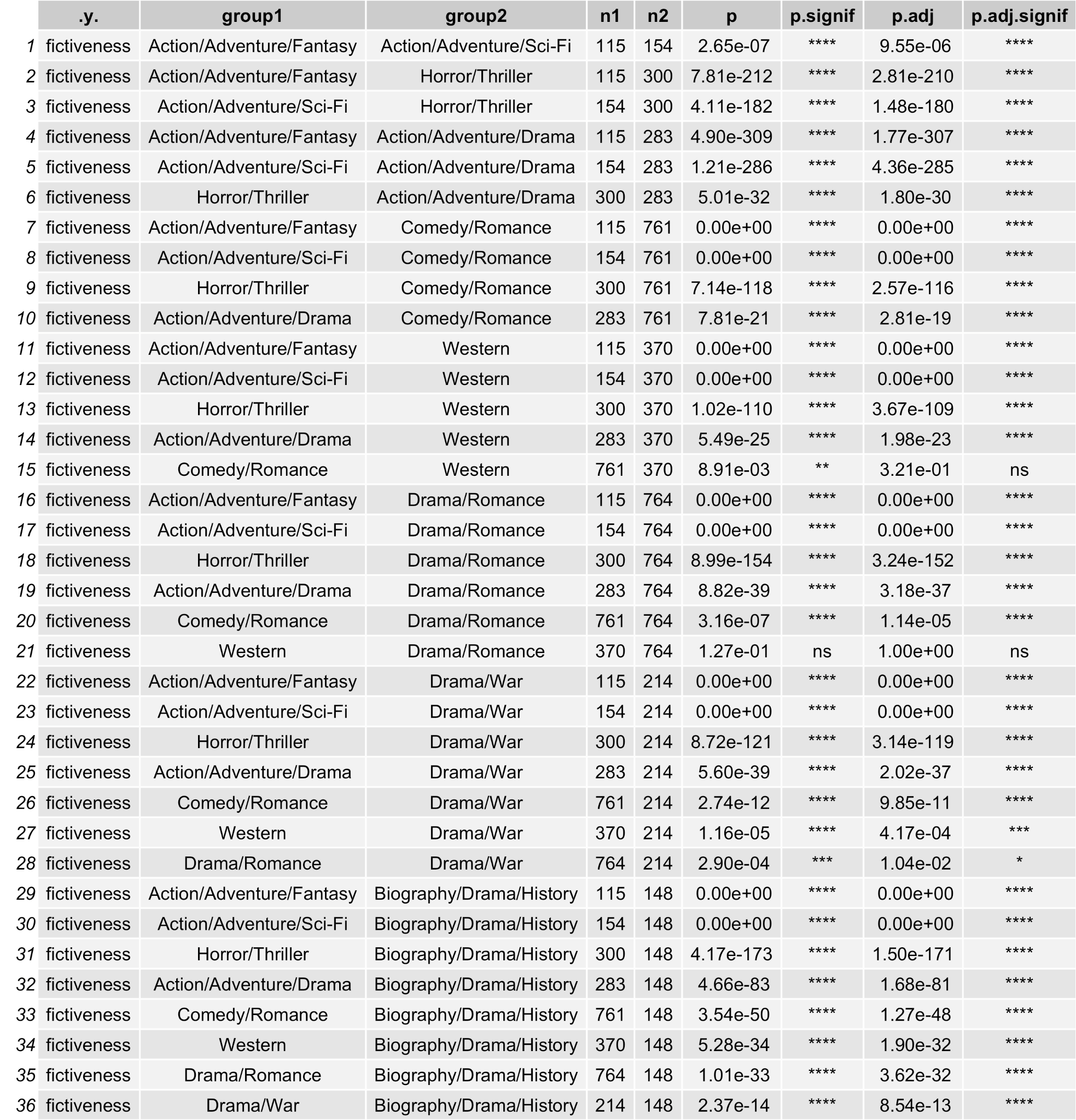
Figure 7. Comparisons of fictiveness between the 9 main genres in IMDb.**

**
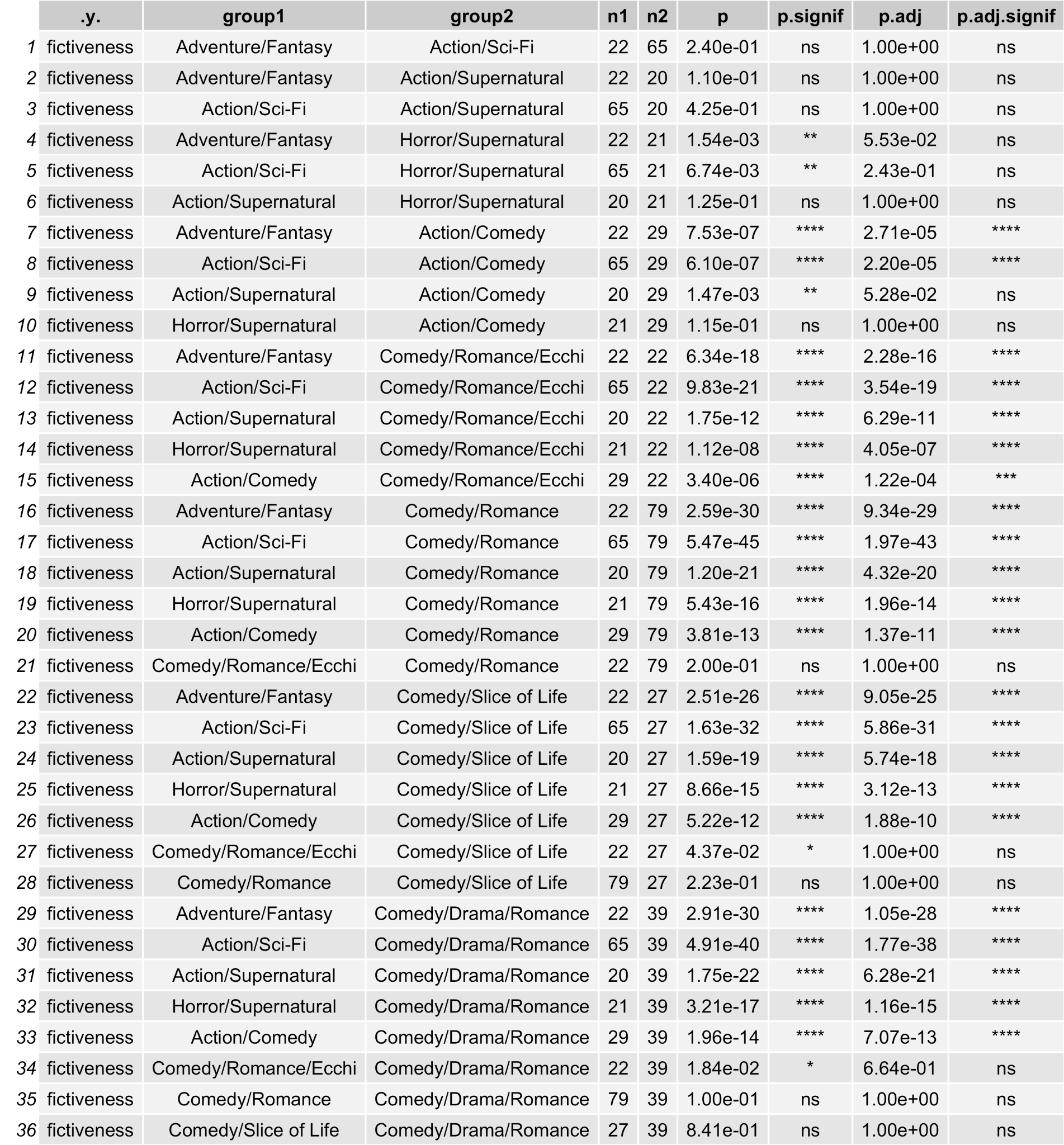
**

**Figure 8. Comparisons of fictiveness between the 9 main genres in MyAnimeList.**
